# Influence of the Drug Position on Bioactivity in Angiopep-2—Daunomycin Conjugates

**DOI:** 10.3390/ijms24043106

**Published:** 2023-02-04

**Authors:** Lilla Pethő, Rita Oláh-Szabó, Gábor Mező

**Affiliations:** 1 ELKH-ELTE Research Group of Peptide Chemistry, 1117 Budapest, Hungary; 2 Institute of Chemistry, Faculty of Science, Eötvös Loránd University, 1117 Budapest, Hungary

**Keywords:** peptide–drug conjugate, Angiopep-2, daunomycin, tumor targeting, bioactivity, structure–activity relationship

## Abstract

The blood–brain barrier (BBB) is a semipermeable system, and, therefore, most of the active substances are poorly transported through this barrier, resulting in decreased therapeutic effects. Angiopep-2 (TFFYGGSRGKRNNFKTEEY) is a peptide ligand of low-density lipoprotein receptor-related protein-1 (LRP1), which can cross the BBB via receptor-mediated transcytosis and simultaneously target glioblastomas. Angiopep-2 contains three amino groups that have previously been used to produce drug–peptide conjugates, although the role and importance of each position have not yet been investigated. Thus, we studied the number and position of drug molecules in Angiopep-2 based conjugates. Conjugates containing one, two, and three daunomycin molecules conjugated via oxime linkage in all possible variations were prepared. The in vitro cytostatic effect and cellular uptake of the conjugates were investigated on U87 human glioblastoma cells. Degradation studies in the presence of rat liver lysosomal homogenates were also performed in order for us to better understand the structure–activity relationship and to determine the smallest metabolites. Conjugates with the best cytostatic effects had a drug molecule at the *N*-terminus. We demonstrated that the increasing number of drug molecules does not necessarily increase the efficacy of the conjugates, and proved that modification of the different conjugation sites results in differing biological effectiveness.

## 1. Introduction

Transporting drugs across the blood–brain barrier (BBB) is a major challenge in the treatment of brain tumors. The BBB is a semipermeable system that separates the peripheral circulation from the extracellular fluid. Its task is to supply the central nervous system with nutrients and oxygen, to set the appropriate ion concentration, and to regulate hormones, as well as to protect the brain against toxic molecules. The actual barrier is formed by the tight junctions of endothelial cells that make up the wall of the brain capillaries, primarily preventing the passage of hydrophilic or high-molecular-weight substances. Hence, the transportation of most active substances through the blood–brain barrier is low, which prevents them from reaching the target cells in the brain and exerting their therapeutic effect there [1]. However, the transport of the molecules needed for brain function is well established. Small hydrophilic molecules (e.g., water and small ions) can pass through the BBB via paracellular transport, while small lipophilic molecules (e.g., hormones, O_2_, CO_2_) pass via transcellular transport. These two processes take place by passive diffusion. Smaller polar building blocks (e.g., glucose, amino acids) are transported with the help of transporter proteins, while macromolecules with high molecular weight or positive charges (e.g., transferrin, albumin, aprotinin) can pass via either adsorption-mediated or receptor-mediated transcytosis [2].

Findings regarding the 19-amino-acid-long Angiopep-2 peptide (TFFYGGSRGKRNNFKTEEY) were first published in 2008. Demeule et al. investigated the passage of the aprotinin protein through the BBB. In order to identify the domain responsible for aprotinin transport, a sequence homology test was performed, and Angiopep-2 was the optimized homologous sequence fragment [3]. This peptide is a ligand for the low-density lipoprotein receptor-related proteins (LRP1 and LRP2) belonging to the low-density lipoprotein receptor (LDLR) family. LRP1 is highly expressed on the endothelial cells of the blood–brain barrier, and is also overexpressed on certain types of tumor cells (e.g., glioblastomas). Thus, LRP1 ligands are first transported to the brain by receptor-mediated transcytosis, and then they selectively enter the tumor cells by receptor-mediated endocytosis. Thereby, dual targeting may be achieved [4].

**Figure 1:**
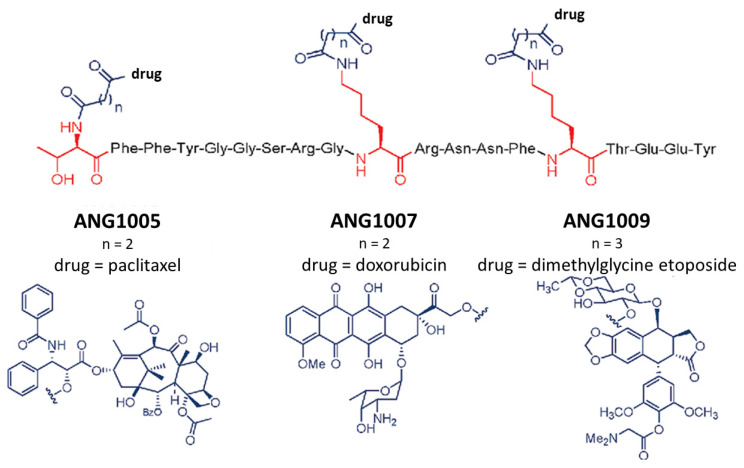
The structure of ANG1005, ANG1007, and ANG1009 [6]. The 3 amino acids that can be used as conjugation sites are marked in red, while the conjugated drugs with the used linkers are represented in grey.

Angiopep-2 was used for the synthesis of peptide–drug conjugates within a short period of time. The first derivative was ANG1005, which contained paclitaxel as the drug molecule. This conjugate could cross the BBB in higher quantities than the free paclitaxel, and its effectiveness against glioblastoma was also successfully demonstrated [5]. In the following years, two more conjugates suitable for directed therapy with similar structures (ANG1007 containing doxorubicin, while ANG1009 containing etoposide) were produced [6]. Angiopep-2 contains 3 amino groups (2 lysine side chains in positions 10 and 15 and the *N*-terminus), and in these studies, all of these groups were functionalized either with glutaric acid or succinic acid to form ester bonds between the peptide and the drug molecules for the development of peptide–drug conjugates (Figure 1).

Using all possible conjugation sites is convenient from a synthetic point of view, and in most cases, more drug molecules result in a higher antitumor effect. However, sometimes the selective substitution of one functional group results in a more effective compound. In case of Angiopep-2, the role and importance of each position have not yet been investigated. Hence, in this work, the number and position of the drug molecules in Angiopep-2-based conjugates were our focus.

## 2. Results

### 2.1. Synthesis of Angiopep-2–Daunomycin Conjugates

**Scheme 1:**
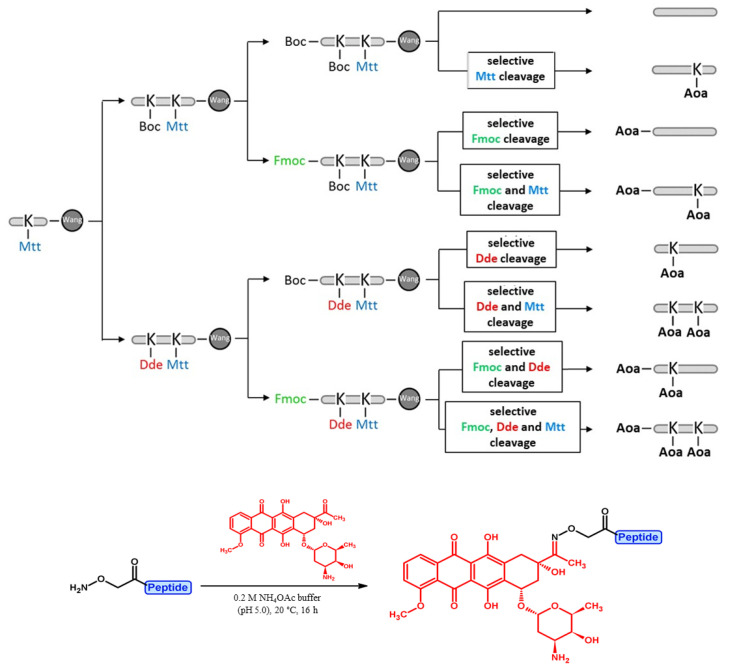
Synthetic protocol of different Angiopep-2–daunomycin conjugates using orthogonal side chain protecting groups (marked with different colors).

The synthesis of the Angiopep-2-based conjugates was carried out on Wang resin, so peptides containing a carboxyl group at the *C*-terminus were obtained after cleavage from the resin. The 19-amino-acid-long peptides were prepared by SPPS using the Fmoc/*t*Bu strategy, starting from one batch of Wang resin. Fmoc-Lys(Mtt)-OH was used as the first lysine derivative (^15^Lys), since the Mtt side chain protecting group can be selectively cleaved on the resin with dilute acid (1–2% TFA/DCM) or it can be removed at the point of cleavage from the resin. In order to avoid a succinimide ring closure reaction, a mixture of 2% piperidine + 2% DBU + 0.1 M HOBt/DMF was used to remove the Fmoc group after the insertion of the second asparagine derivative (^12^Asn). Before the coupling of the second lysine derivative (^10^Lys), the resin was split (1:1 ratio), and orthogonal protecting schemes were further applied to create the proper conjugation sites (Scheme 1). Namely, Fmoc-Lys(Dde)-OH containing a selectively removable side chain protecting group was incorporated in case of compounds **4** and **6**–**8**, while the standard Fmoc-Lys(Boc)-OH was used in case of compounds **1**–**3** and **5**. Before the coupling of the last amino acid, the resins were split again (1:1 ratio, both), and either Fmoc-Thr(*t*Bu)-OH (for compounds **3**, **5**, **7**, and **8**) or Boc-Thr(*t*Bu)-OH (for compounds **1**, **2**, **4**, and **6**) was introduced to the peptide chain. Before the cleavage of the peptides from the resin, one or more protecting groups were removed selectively, and the resulting free amino groups were modified by Boc-protected aminooxyacetyl (Aoa) moiety. After the peptide cleavage, when free aminooxyacetic acid was added as a ‘carbonyl capture’ reagent to prevent the unwanted side reaction of the aminooxyacetylated peptide with ketones or aldehydes [7], the derivatives containing different numbers of Aoa were purified by RP-HPLC and used for conjugation with daunomycin (Dau) under slightly acidic conditions (0.2 M NH_4_OAc at pH 5.0, overnight). In this way, three conjugates with one Dau in different positions, three conjugates with two Dau, and one with three Dau in each possible conjugation site were obtained. Analytical HPLC chromatograms and MS spectra of Angiopep-2 peptide and daunomycin-Angiopep-2 conjugates are shown in the Supplementary Materials (Table S1 and Figures S1–S8).

### 2.2. In Vitro Cytostatic Assays of Angiopep-2–Daunomycin Conjugates

The in vitro cytostatic effect of the synthesized peptide (**1**) and daunomycin-peptide conjugates (**2**–**8**) was investigated on U87 human glioblastoma cells. The cells were treated with the peptide and conjugates at different concentrations (0.05–50 μM) for 24 h, and after a washing step, the cells were incubated for another 48 h at 37 °C. The cytostatic effect of the compounds was determined using the MTT test. The measured IC_50_ values are shown in Table 1.

The free Angiopep-2 did not show any effect on tumor cells up to a 50 µM concentration. However, no clear correlation could be obtained between the number of the daunomycin and the cytostatic efficacy. Among the conjugates with only one Dau, compound **3,** in which Dau was attached to the *N*-terminus, showed the highest in vitro cytostatic effect on glioblastoma cells. When the Dau was conjugated to the Lys side chain in position 15 (compound **2**), the effect decreased significantly, and conjugate **4** was also not significantly better. In case of conjugates with two drug molecules, a similar tendency was observed. Compound **7,** with Dau at the *N*-terminus and on the Lys side chain in position 10, showed the highest activity. When the Lys side chains were used as conjugation sites, the formed compound (**6**) had moderate activity on U87 cells. Surprisingly, conjugate **5,** with Dau at the *N*-terminus and Lys side chain in position 15, showed the lowest efficacy on the U87 cells. Compound **8,** with three Dau, showed moderate activity. Thus, the effectiveness of Angiopep-2–daunomycin conjugates does not primarily depend on the number of drug molecules, but rather on their position within the molecule.

The data indicated that the side chain of Lys in position 15 is not a good conjugation site in terms of the cytostatic effect and drug design. In contrast, the substitution of the *N*-terminus amino group with a drug molecule might be the most efficient choice for drug targeting with Angiopep-2.

### 2.3. In Vitro Cellular Uptake Studies of Angiopep-2–Daunomycin Conjugates

**Figure 2:**
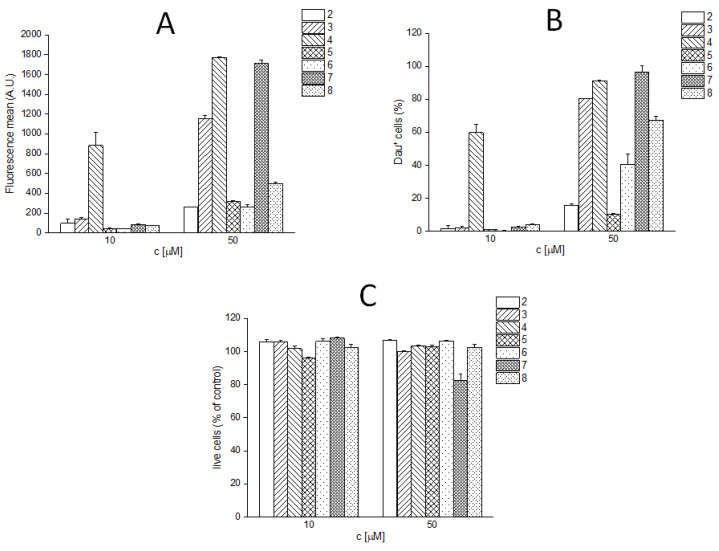
In vitro cellular uptake of Angiopep-2–daunomycin conjugates at 10 and 50 µM. (**A**) fluorescence mean; (**B**) percentage of the Dau-positive live cells (cells showing fluorescence intensities above the highest fluorescence intensity value of the untreated control cells were considered as Dau+); (**C**) live cells in the percentage of untreated control.

The in vitro cytostatic effect of any peptide–drug conjugate is influenced by many factors. One of the main effects is the cellular uptake of the conjugates by cells. Therefore, we compared the cellular uptake of the different conjugates on U87 glioblastoma cells. The cells were incubated with the conjugates (2, 10, and 50 µM) for 1 h. Then, after a washing step, the cells were treated with trypsin to remove the cell surface-bound conjugates and other cell surface structures that enable non-specific binding, so only the quantity of the conjugates actually entering the cells was detected by flow cytometry. In all cases, the fluorescence intensity values showed a good correlation with the percentage of daunomycin-positive cells (Figure 2A,B). In the case of one conjugate (**7**), a slight toxicity was observed during the measurement at a concentration of 50 µM (Figure 2C). The cellular uptake of the conjugates was concentration-dependent. The correlation between the cellular uptake and the cytostatic effect could be established in most cases. Conjugates **3** and **7**, which showed the highest cytostatic effect, entered the cells very efficiently at a concentration of 50 µM (80% and 97% of cells were Dau+, respectively), while conjugate **2** showed very low uptake (15%) (Figure 2A,B). It seems that the slightly, but not significantly, higher cytostatic effect of conjugate **7** is related mainly to the elevated cellular uptake induced by the substituent on the side chain of ^10^Lys, but not because of the two drug molecules. Interestingly, conjugate **4**, which showed moderate cytostatic effect, was taken up the most effectively: 90% of the cells were Dau+ at a concentration of 50 µM, while this value was 60% at a concentration of 10 µM. In contrast, all other compounds showed lower cellular uptake (<10%) at this concentration. In case of the conjugates **5** and **6**, as well as the conjugate with 3 Dau (**8**), only 65–70% of the cells were Dau+ at a concentration of 50 µM. Presumably, the substitution of the side chain of Lys in position 15 with a drug molecule (**2**, **5**, **6**, and **8**) significantly reduces the receptor binding of these conjugates, and, consequently, their cellular uptake. Thus, this low internalization ability is responsible for their lower in vitro cytostatic effect.

### 2.4. Degradation of Daunomycin–Angiopep-2 Conjugates in Rat Liver Lysosomal Homogenate

Since we discovered contradictions between the cellular uptake and cytostatic effect of conjugate **4**, we studied the degradation of the conjugates in lysosomal homogenates (isolated from rat livers).

**Figure 3:**
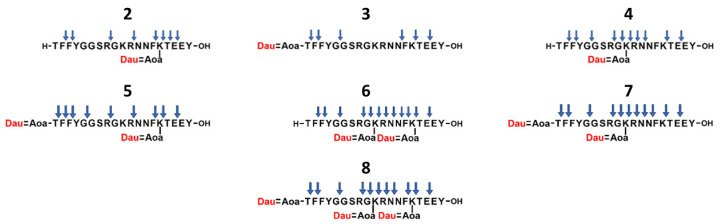
The identified cleavage sites (indicated by blue arrows) of the Angiopep-2–daunomycin conjugates in lysosomal homogenate. The position of the drug molecule is marked with red.

The conjugates were treated with lysosomal homogenate for 5 min, 1 h, 6 h, 24 h, and 72 h, and the degradation was followed by HPLC-MS. The identified cleavage sites are shown in Figure 3. Control studies investigating conjugates in buffer (without lysosomal homogenate) were also performed, and for these, all conjugates were stable. The results of the degradation indicated that in the case of compound **3**, the Dau=Aoa-Thr-OH fragment appeared within 1 h. At 6 h, it was the main drug-containing metabolite, and at 72 h, only this Dau-containing fragment could be detected. This time-dependent pattern was also observed in the case of conjugate **2**, where Dau was attached to the side chain of Lys in position 15, and the formed smallest metabolite was H-Lys(Dau=Aoa)-OH. In contrast, this metabolite was not efficiently released from compound **4**. It was not detectable at 1 h, and even after 72 h, the main daunomycin-containing metabolites were H-Gly-Lys(Dau=Aoa)-Arg-OH and H-Gly-Lys(Dau=Aoa)-OH. It is likely that, in this case, a large steric hindrance occurred, and the enzymes were not able to access the peptide bonds around ^10^Lys. This observation might explain the discrepancy between the efficient cellular uptake and the lower anticancer activity of this conjugate. Hence, if the drug molecule is conjugated through a non-enzyme-cleavable linker, special attention must be paid to the selection of the conjugation site, since the mechanism of degradation of the conjugate can significantly influence its biological effect.

The detected total ion chromatograms and the identified degradation fragments are given in the Supplementary Materials (Figures S9–S15 and Tables S2–S8).

### 2.5. Preparation of Conjugates Containing an Enzyme-Labile Spacer

Since the cellular uptake of compound **4** was outstanding, Cathepsin B (lysosomal enzyme) labile peptide spacers were incorporated between the side chain of Lys in position 10 and Dau to increase the release of the active metabolite in this position. For this purpose, GFLG [8] and VA [9] were selected. In case of the Val-Ala sequence, the lysosomal enzymes first cleave after the alanine, which, in this case, would be connected to the lysine side chain by an isopeptide bond that usually cannot be cleaved by enzymes. Thus, a spacer containing two additional glycines as linkers between the alanine and the ε-amino group (VAGG) was also designed.

Peptides were synthesized as described above, starting from one batch of Wang resin. Fmoc-Lys(Boc)-OH was used in position 15 and Fmoc-Lys(Dde)-OH in position 10, while Boc-Thr(*t*Bu)-OH was used as the last amino acid. After the synthesis of the peptide backbone, the Dde-protecting group was cleaved, the resin was split (1:1:1 ratio) and the enzyme-labile spacers were built on the lysine side chain. Finally, Boc-Aoa-OH was coupled to all derivatives. The peptides were cleaved from the resin and purified by RP-HPLC, and then the fraction containing the pure product was used for the drug conjugation. Analytical HPLC chromatograms and MS spectra of the enzyme labile spacer containing Angiopep-2–daunomycin conjugates are shown in the Supplementary Materials (Table S9 and Figures S16–S18).

### 2.6. In Vitro Cytostatic Assay and In Vitro Cellular Uptake Studies of the Enzyme-Labile Spacer Containing Angiopep-2–Daunomycin Conjugates

**Figure 4:**
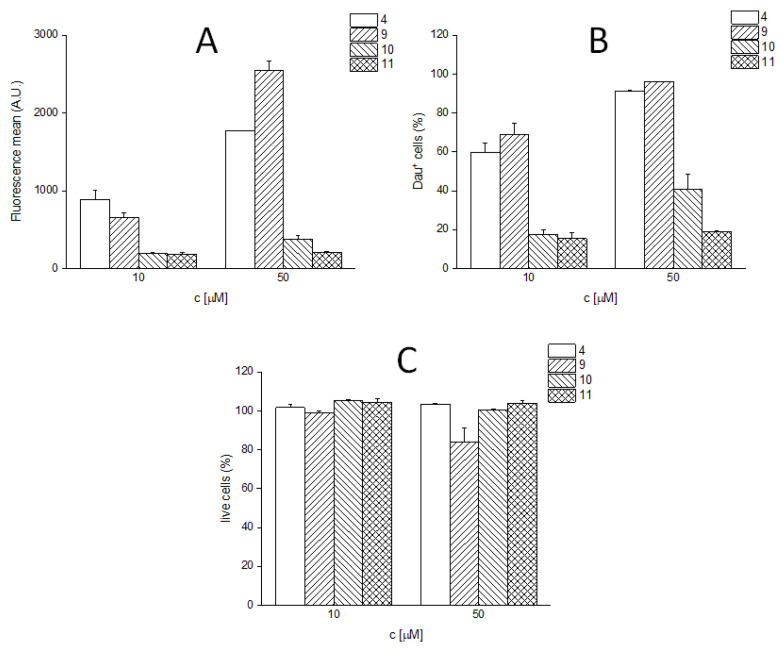
In vitro cellular uptake of the enzyme-labile spacer containing Angiopep-2–daunomycin conjugates. (**A**) Fluorescence mean; (**B**) percentage of Dau-positive live cells (cells showing fluorescence intensities above the highest fluorescence intensity value of the untreated control cells were considered as Dau+); (**C**): live cells in the percentage of untreated control.

The in vitro cytostatic effect and the in vitro cellular uptake of the spacer-containing daunomycin–peptide conjugates (**9–11**) were studied on U87 human glioblastoma cells as previously described. The measured IC_50_ values are shown in Table 2, while the measured cellular uptake at 10 µM and 50 µM concentration are given in Figure 4. The fluorescence intensity values showed good correlation with the percentage of daunomycin-positive cells in the case of these conjugates as well. (Figure 4A,B). Conjugate **9** showed slight toxicity during the measurements at a concentration of 50 µM (Figure 4C).

With the incorporation of the VA spacer alone (**10**), the cytostatic effect was completely lost, while the other two derivatives (**9** and **11**) showed efficacy similar to that of the parent conjugate (**4**). The cellular uptake of the conjugate containing the GFLG spacer (**9**) was comparable to that of conjugate **4**, while the incorporation of VA (**10**) and VAGG (**11**) spacers significantly decreased the cellular uptake.

### 2.7. Degradation of the Enzyme-Labile Spacer Containing Angiopep-2–Daunomycin Conjugates in Rat Liver Lysosomal Homogenate

**Figure 5:**
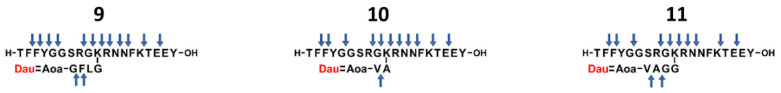
The identified cleavage sites (indicated by blue arrows) of the enzyme-labile spacer containing Angiopep-2–daunomycin conjugates in lysosomal homogenate.

The degradation studies of the enzyme-labile spacer containing conjugates in lysosomal homogenate were performed as described previously, and the identified cleavage sites are shown in Figure 5. The detected total ion chromatograms and the identified degradation fragments are given in Supplementary Materials (Figures S19–S21 and Tables S10–S12).

As expected, the VA spacer alone (compound **10**) inhibited the enzymatic cleavage because of the isopeptide bond between the alanine and the lysine side chain, and the expected metabolite (Dau=Aoa-Val-OH) was only produced in negligible amounts after 72 h. Furthermore, H-K(Dau=Aoa-VA)-OH was also only formed in very small amounts, probably due to steric hindrance, as observed at compound **4**. In this case, the loss of the in vitro cytostatic effect may be explained both by the decrease in cellular uptake and the inhibited lysosomal degradation. The incorporation of two Gly between VA and the Lys side chain (compound **11**) significantly changed the degradation pattern. The Dau=Aoa-Val-Ala-OH fragment appeared after 1 h, and Dau=Aoa-Val-OH was also detected in high amounts after 6 h. This could indicate high antitumor activity, but its cytostatic effect was comparable to that of compound **4**, which may be explained by the low in vitro cellular uptake of this conjugate. In the case of the GFLG spacer in conjugate **9**, the release of Dau=Aoa-Gly-OH was particularly slow, and the detected main metabolite at the 6 h time point was Dau=Aoa-Gly-Phe-OH. Even after 24 h, one of the most intensively detectable fragments was H-GK(Dau=Aoa-GFLG)R-OH, so it is very likely that steric inhibition also occurred in this case, resulting in a similar in vitro cytostatic effect to that of compound **4**.

## 3. Discussion

Several Angiopep-2 conjugates were developed after the first successful attempts of ANG1005, ANG1007, and ANG1009. In the case of ANG2002, neurotensin was linked via a *C*-terminal cysteine incorporated into Angiopep-2 to produce a pain reliever acting in the central nervous system [10]. In the case of ANG4043, four Angiopep-2 peptides were linked via their *N*-terminus to a HER2-selective monoclonal antibody for the treatment of brain metastases of breast cancer [11]. In addition, several other papers have been published on the conjugation of Angiopep-2 to nanoparticles (NPs) [12,13,14,15,16,17,18,19,20,21,22,23,24] wherein the peptide was coupled, via a cysteine incorporated to the *C*-terminus (thioether bond), to the various NPs. The in vitro and in vivo studies showed that all NPs modified with Angiopep-2 were able to target brain tumor cells selectively and efficiently.

The antitumor effect of peptide–drug conjugates strongly depends on the targeting peptide, the number of drugs that can be maximally released from the conjugate, the cellular uptake, and its degradation within the cell. It is also important to choose the correct conjugation site for the drug molecule, which can reduce receptor binding through steric hindrance or change the structure of the conjugate, thus inhibiting or slowing down the release of the active metabolite. During the design of the conjugates, these aspects must be taken into account in every case; these effects add up and, together, they can influence the antitumor effect in a negative direction.

In the case of Angiopep-2, the role and importance of the three amino groups (the *N*-terminus and the ^10^Lys and ^15^Lys side chain amino groups) have not yet been investigated. In all conjugates containing a small drug molecule, all three amino functional groups were applied for drug conjugation using ester bonds. Nevertheless, this type of linker is not fully stable in the bloodstream; thus, in in vivo experiments, the application of esterase inhibitors is suggested. In addition, in a previous study, we detected that *O*→*N* shifts (to the aminosugar moiety) can happen in cases where ester-linked doxorubicin is present during the working-up and storage procedures [25]. This observation can be proven by ESI-MS, where different fragments of the conjugates can be observed under normal conditions of the analysis due to the lability of the *O*-glycosidic bond [26]. In contrast, the oxime-linked peptide–daunomycin conjugates can be prepared easily in high amounts by this click-type reaction. It is also worth mentioning that oxime linkage is quite stable at a neutral pH; therefore, there is no early release of the drug from the conjugate into blood circulation, in contrast with conjugates containing an ester linkage (e.g., ANG1005). The main disadvantage of the oxime linkage is that it does not decompose in cells either to release the free drug. However, the smallest metabolites that contain daunomycin (Dau linked to one amino acid through the Aoa linker) can bind to DNA, resulting in antitumor activity [27]. For that reason, oxime-linked conjugates are suitable for searching for appropriate homing peptides for the further development of drug delivery systems. In addition, this is the reason why this type of conjugate can be used in higher amounts in vivo; they provide a greater antitumor effect in comparison with free Dau at the maximum tolerated dose [28,29,30].

In this study, we made systematic comparisons of Angiopep-2–daunomycin conjugates with different numbers and payload positions. The results of the in vitro cytostatic assay on U87 human glioblastoma cells show that the most efficient conjugates were compound **3**, containing only one daunomycin at the *N*-terminus; and compound **7**, containing Dau at the *N*-terminus and on the side chain of **^10^**Lys. However, further increasing the number of the active ingredients (i.e., using all possible conjugation sites) does not improve, but rather, decreases the efficiency of the conjugates. In addition, the substitution of the side chain of **^10^**Lys or **^15^**Lys, or of both positions, results in conjugates with moderate efficacy. In order to better understand the structure–activity relationship (SAR), the cellular uptake by U87 cells and the lysosomal degradation of the conjugates were investigated. The conjugate containing only one daunomycin at the *N*-terminus (compound **3**) had the second-best cytostatic effect, and also had a favorable in vitro cellular uptake and lysosomal degradation profile. However, the modification of the lysine closer to the *C*-terminus (^15^Lys) significantly decreased the efficiency despite the fast release of the active metabolite, which can be attributed to the low cellular uptake. By substituting the lysine closer to the *N*-terminus (^10^Lys), a conjugate with outstanding internalization ability was produced, but its cytostatic efficiency was moderate. It became obvious from the degradation studies that the slow metabolism caused the low cytostatic activity. This may be overcome with an enzyme-labile spacer between the drug molecule and the lysine side chain; however, a properly designed structure and spacer sequence is required to control all emerging aspects. In the case of conjugates with more than one Dau, additive effects were observed in the cellular uptake studies. These are also related to changes in the cytostatic effect.

From our results, we can conclude that it is not worth attaching any compounds to the side chain of ^15^Lys. Furthermore, the substitution of the side chain of ^10^Lys with a drug molecule without a spacer or with a non-degradable linker is not suggested, especially if the release of the payload is necessary for its biological activity. Finally, one drug molecule attached to the *N*-terminus might be as good or even better than conjugates with three drug molecules. Therefore, the easier, synthetic way to substitute all conjugation sites of Angiopep-2 should not be a reason to develop potent peptide–drug conjugates. Consequently, it is unambiguous that the position of the drug molecule in an Angiopep-2 conjugate has a greater influence on the antitumor activity than the number of conjugated drugs.

## 4. Materials and Methods

### 4.1. Materials

All amino acid derivatives, the Wang resin, *N*,*N*′-diisopropylcarbodiimide (DIC), and trifluoroacetic acid (TFA) were obtained from Iris Biotech GmBH (Marktredwitz, Germany). 1-hydroxybenzotriazole hydrate (HOBt), Boc-aminooxyacetic acid (Boc-Aoa-OH), 4-dimethylaminopyridine (DMAP), triisopropylsilane (TIS), diisopropylethylamine (DIPEA), and hydrazine hydrate were purchased from Sigma Aldrich Kft (Budapest, Hungary). Aminooxyacetic acid (H-Aoa-OH·1/2HCl) and 1,8-diazabicyclo [5.4.0]undec-7-ene (DBU) were obtained from TCI Europe N.V. (Zwijndrecht, Belgium), (benzotriazol-1-yloxy) tris (pyrrolidino) phosphonium hexafluorophosphate (PyBOP) from Bachem AG (Bubendorf, Switzerland), the activating reagent ethyl 2-cyano-2-(hydroxyimino)acetate (Oxyma Pure) from Novabiochem (Darmstadt, Germany), and piperidine from Molar Chemicals Kft (Budapest, Hungary). Daunomycin hydrochloride was a gift from IVAX (Budapest, Hungary). The solvents used during synthesis and purification (*N*,*N*-dimethylformamide (DMF), dichloromethane (DCM), ethanol, diethyl ether, acetonitrile, acetone) and the ammonium acetate (NH_4_OAc) salt used as a buffer were all obtained from VWR International Kft (Debrecen, Hungary).

For the in vitro biological assays, DMEM medium, L-glutamine, penicillin–streptomycin antibiotic mixture, sodium pyruvate, non-essential amino acid mixture, HPMI, and trypsin were obtained from Lonza Group AG (Basel, Switzerland), FBS was purchased from BioSera Europe (Nuaille, France), and dimethyl sulfoxide (DMSO) and MTT solution were obtained from Merck KGaA (Darmstadt, Germany). The 96-well tissue culture plates were purchased from Sarstedt AG & Co. KG (Nümbrecht, Germany), and the 24-well tissue culturing plates from Greiner Bio-One AG (Kremsmünster, Austria).

The solvents and sodium acetate salt used during the mass spectrometric measurements were purchased from Sigma Aldrich Kft (Budapest, Hungary), and in all cases, they were of analytical grade or of the highest purity available. Double-distilled water was used in all cases for sample preparation.

### 4.2. Peptide Synthesis

Angiopep-2 derivatives were synthesized by SPPS on Wang resin (0.45 mmol/g resin capacity) using the Fmoc/*t*Bu strategy. The coupling of the first amino acid (Fmoc-Tyr(tBu)-OH; 2 eq) was carried out for 2 h with DIC in the presence of DMAP (2:0.2 eq). All other amino acids were coupled with DIC and HOBt (3:3:3 eq). The Fmoc group was removed with a cleavage mixture of 2% piperidine + 2% DBU/DMF, which was changed to 2% piperidine + 2% DBU + 0.1 M HOBt/DMF mixture after the coupling of the second asparagine (^12^Asn) to prevent the succinimide formation.

The peptides were synthesized starting from one batch of resin. In the case of the first lysine (^15^Lys), Fmoc-Lys(Mtt)-OH was coupled. Before the coupling of the second lysine derivative, the resin was split (1:1 ratio), and orthogonal protecting schemes were further applied to create the proper conjugation sites: Fmoc-Lys(Boc)-OH to compounds **1–3** and **5** and Fmoc-Lys(Dde)-OH to compounds **4** and **6–8**. Before the coupling of the last amino acid derivative, the resins were divided again (1:1 ratio, both). In the case of the sequences containing a free amino group at the *N*-terminus (**1**, **2**, **4**, and **6**), Boc-Thr(*t*Bu)-OH was introduced to the peptide chain, while in the other cases (**3**, **5**, **7**, and **8**), Fmoc-Thr(*t*Bu)-OH was used. After the synthesis of the peptide’s backbone, the appropriate *N*-terminus and/or lysine side chain-protecting groups were selectively cleaved (Fmoc: 2% piperidine + 2% DBU + 0.1 M HOBt/DMF, Dde: 2% N_2_H_4_·H_2_O/DMF, Mtt: 1% TFA/DCM). Then, Boc-protected aminooxyacetic acid (Boc-Aoa-OH) was coupled with PyBOP, Oxyma Pure, and DIPEA (5:5:5:10 eq calculated for the free amino group). The peptides were cleaved from the resin with a mixture of 95% TFA, 2.5% TIS, and 2.5% distilled water in the presence of 10 eq of free aminooxyacetic acid as a ‘carbonyl capture’ reagent to prevent the undesired side reaction of the aminooxyacetylated peptide. The crude peptides were purified by RP-HPLC (KNAUER RP-HPLC, Knauer GmbH, Bad Homburg, Germany; Phenomenex Luna C18 column (10 μm, 100 Å, 250 × 21.2 mm), Torrence, CA, USA) and the products were identified by mass spectrometry (ESI-MS Bruker Daltonics Esquire 3000 Plus, Bremen, Germany).

The peptides for compounds **9**–**11** were synthesized similarly, but Fmoc-Lys(Boc)-OH was used as the first lysine (^15^Lys), while Fmoc-Lys(Dde)-OH was coupled as the second lysine (^10^Lys) and Boc-Thr(*t*Bu)-OH was introduced as the last amino acid (^1^Thr). After the synthesis of the peptide’s backbone, the Dde side chain-protecting group was cleaved, the resin was split into three portions (1:1:1 ratio), and the spacer sequences were built on the ε-amino group of ^10^Lys. Boc-Aoa-OH was coupled as the last unit to each derivative. Peptides were cleaved from the resin and then processed as previously described.

### 4.3. Synthesis of the Angiopep-2–Daunomycin Conjugates

The HPLC fractions containing the pure peptides were always used directly (after concentration, but without lyophilization) for the conjugation of daunomycin. Next, 1.5 eq daunomycin·HCl (calculated to the Aoa groups) was added and the pH was adjusted with ammonium acetate buffer (0.2 M, pH 5.0; 10 mg/mL peptide concentration). The reactions were stirred at room temperature for 16 h, then purified by RP-HPLC, and the resulting conjugates were identified by mass spectrometry.

### 4.4. Cells and Culturing

U87 human glioblastoma cell line [31] was cultured in DMEM supplemented with 10% FBS, 2 mM L-glutamine, penicillin–streptomycin antibiotic mixture (50 IU/mL and 50 μg/mL, respectively), 1 mM sodium pyruvate, and 1% non-essential amino acid mixture. The cell culture was maintained at 37 °C in a humidified atmosphere with 5% CO_2_. The U87 cell line was a generous gift of Dr. József Tóvári (Department of Experimental Pharmacology, National Institute of Oncology, Budapest, Hungary).

### 4.5. In Vitro Cytostasis Assays

For determining the cytostatic effect of the conjugates, U87 cells were grown to confluency and then divided into 96-well tissue culture plates with an initial cell number of 5.0 × 10^3^ cells/well. After 24 h incubation at 37 °C, the cells were treated with the compounds overnight in the concentration range of 0.05–50 μM in 200 μL final volume containing 1.0 *v/v*% DMSO, whereas control cells were treated only with serum-free medium or with 1.0 *v/v*% DMSO at the same conditions. After the incubation, cells were washed twice with serum-free medium, then the cells were cultured for an additional 72 h in 10% serum-containing medium at 37 °C. Following that, MTT solution (at c = 0.37 mg/mL final concentration) was added to each well. The respiratory chain [32] and other electron transport systems [33] reduced MTT and thereby formed non-water-soluble violet formazan crystals within the cells [34]. The amount of these crystals can be determined by spectrophotometry and serves as an estimate for the number of mitochondria in addition to the number of living cells in the well [35]. After 3 h of incubation with MTT, the cells were centrifuged for 5 min at 2000 rpm and the supernatant was removed. The obtained formazan crystals were dissolved in DMSO (100 µL), and the optical density (OD) of the samples was measured at λ = 540 nm and 620 nm, respectively, using ELISA Reader (iEMS Reader, Labsystems, Finland). The OD_620_ values were subtracted from the OD_540_ values. The percent of cytostasis was calculated with the following equation:

(1) $$\mathrm{Cytostatic}\;\mathrm{effect}\;(\%)=1-\frac{{\mathrm{OD}}_{\mathrm{treated}}}{{\mathrm{OD}}_{\mathrm{control}}}\times 100$$

where the OD_treated_ and OD_control_ values correspond to the optical densities of the treated and the control wells, respectively. In each case, two independent experiments were carried out with four parallel measurements. Cytostasis was plotted as a function of concentration, and the half-maximal inhibitory concentration was calculated based on a sigmoid curve fitted on the data points using Microcal™ Origin 2018 software. IC_50_ represents the concentration of a compound that is required for 50% inhibition, expressed in micromolar units.

### 4.6. In Vitro Cellular UPTAKE studies

U87 cells were plated into 24-well tissue culturing plates with the initial cell number of 10^5^ cells/well 24 h before the experiment. Cells were washed first with serum-free medium and then incubated with the compounds dissolved in serum-free medium at 2 µg/mL, 10 µg/mL, and 50 µg/mL concentrations for 1.5 h at 37 °C. Control cells were treated with serum-free medium in the same conditions. Following that, cells were washed with HPMI (9 mM glucose, 10 mM NaHCO**_3_**, 119 mM NaCl, 9 mM HEPES, 5 mM KCl, 0.85 mM MgCl**_2_**, 0.53 mM CaCl**_2_**, 5 mM Na**_2_**HPO**_4_** × 2H**_2_**O, pH 7.4) harvested with 0.5 g/L trypsin (treated for 10 min). Trypsinization was stopped using HPMI supplemented with 10% FBS. Cells were centrifuged at 1000 rpm for 5 min and the pellet was resuspended in HPMI. The cells were examined with a BD LSR II flow cytometer (Beckton Dickinson, Franklin Lakes, NJ, USA). Data were recorded for 5000–10,000 cells at λ_ex_ = 488 nm. The fluorescence mean was calculated. Daunomycin-positive cells were also gated from the living cell population, and the percentage of daunomycin-containing cells was calculated. The gating was determined from the untreated control cell count/fluorescence intensity histogram by adjusting the lowest fluorescence intensity value of the gated cells to the highest fluorescence intensity value of the live population of untreated control cells. Cells showing fluorescence intensities above this were considered as Dau-positive. Statistical analysis of the data was performed using Student’s *t*-test at a 95% confidence level.

### 4.7. Stability Tests in Rat Liver Lysosomal Homogenates

A stock solution with a concentration of 2.5 μg/μL was prepared from the conjugates in distilled water, and this was diluted 100× with 0.2 M sodium acetate buffer (pH 5.03). The rat liver lysosomal homogenate (16.6 μg/μL) was diluted 20× with the same buffer, and then the two solutions were mixed in a 1:1 (protein:peptide, m/m) ratio. In each case, control measurements were also performed, in which buffer was added to the conjugates instead of the lysosomal homogenate. The solutions were stirred at 600 rpm at 37 °C in a thermostat (ThermoMixer, Eppendorf AG, Hamburg, Germany), and after 5 min, 1 h, 6 h, 24 h, and 72 h, 50 μL samples were taken. Each reaction was quenched with 5 μL formic acid and the samples were stored frozen until analysis.

The HPLC-MS analysis of the samples was performed with a Dionex 3000 HPLC-Q Exactive Focus hybrid quadrupole-orbitrap mass spectrometer (Thermo Fisher Scientific, Bremen, Germany). For separation, a Supelco Ascentis C18 column (3 μm, 100 Å, 150 × 2.1 mm; Merck, Darmstadt, Germany) and gradient elution (0 min 2% B, 1 min 2% B, 17 min 90% B; A eluent: 0.1% formic acid/distilled water, eluent B: 0.1% formic acid/acetonitrile + distilled water (80:20, *v/v*%), flow rate 0.2 mL/min, 40 °C) was used, and high-resolution mass spectra were recorded in the range of 200–1800 *m/z*. The data were analyzed using the Xcalibur program (Thermo Fisher Scientific).

## Figures and Tables

**Table 1:** The measured IC_50_ values of the free Angiopep-2 peptide and the Angiopep-2–daunomycin conjugates.

Code	Compound	IC_50_/µM
1	H-TFFYGGSRGKRNNFKTEEY-OH	>50
2	H-TFFYGGSRGKRNNFK(Dau=Aoa)TEEY-OH	30.2 ± 6.4
3	Dau=Aoa-TFFYGGSRGKRNNFKTEEY-OH	10.9 ± 2.8
4	H-TFFYGGSRGK(Dau=Aoa)RNNFKTEEY-OH	24.0 ± 5.9
5	Dau=Aoa-TFFYGGSRGKRNNFK(Dau=Aoa)TEEY-OH	32.3 ± 8.1
6	H-TFFYGGSRGK(Dau=Aoa)RNNFK(Dau=Aoa)TEEY-OH	21.6 ± 5.4
7	Dau=Aoa-TFFYGGSRGK(Dau=Aoa)RNNFKTEEY-OH	7.8± 6.3
8	Dau=Aoa-TFFYGGSRGK(Dau=Aoa)RNNFK(Dau=Aoa)TEEY-OH	23.6 ± 6.3

**Table 2:** The measured IC_50_ values of the enzyme-labile spacer containing Angiopep-2–daunomycin conjugates.

Code	Compound	IC_50_/µM
4	H-TFFYGGSRGK(Dau=Aoa)RNNFKTEEY-OH	24.0 ± 5.9
9	H-TFFYGGSRGK(Dau=Aoa-GFLG)RNNFKTEEY-OH	16.9 ± 5.6
10	H-TFFYGGSRGK(Dau=Aoa-VA)RNNFKTEEY-OH	>50
11	H-TFFYGGSRGK(Dau=Aoa-VAGG)RNNFKTEEY-OH	20.2 ± 3.0

## Data Availability

The data presented in this study are available in the Supplementary Materials.

## Supplementary Materials

The following supporting information can be downloaded at: https://www.mdpi.com/article/10.3390/ijms24043106/s1.
