# Supplementary material for: Influence of the Drug Position on Bioactivity in Angiopep-2—Daunomycin Conjugates
Source: Int J Mol Sci. 2023 Feb 4;24(4):3106. doi: 10.3390/ijms24043106 (PMC9959518; doi:10.3390/ijms24043106)
Supplement: Supplementary file 1 [file ijms-24-03106-s001.zip › ijms-2132654-supplementary.pdf]

Influence of the Drug Position on Bioactivity in Angiopep-2—Daunomycin Conjugates

Lilla Pethő [1], Rita Oláh-Szabó [1,†] and Gábor Mező [1,2,*]

[1] ELKH-ELTE Research Group of Peptide Chemistry, 1117 Budapest, Hungary
[2] Institute of Chemistry, Faculty of Science, Eötvös Loránd University, 1117 Budapest, Hungary
* Correspondence: gabor.mezo@ttk.elte.hu
† Present address: Department of Genetics, Cell- and Immunobiology, Faculty of Medicine, Semmelweis University, 1089 Budapest, Hungary.

Table of contents

Analytical data of compounds **1-8** 2

Analytical chromatograms and MS spectra of compounds **1-8** 3

Total ion chromatograms of the lysosomal degradation of compounds **1-8** 7

Detected fragments of the lysosomal degradation of compounds **1-8** 11

Analytical data of compounds **9-11** 17

Analytical chromatograms and MS spectra of compounds **9-11** 17

Total ion chromatograms of the lysosomal degradation of compounds **9-11** 19

Detected fragments of the lysosomal degradation of compounds **9-11** 21

Table S1. Analytical data of the synthesized Angiopep-2 peptide and the daunomycin-Angiopep-2 conjugates

Code	Compound	R_t / min[a]	MW_{calc} / MW_{meas}[b]
**1**	H-TFFYGGSRGKRNNFKTEEY-OH	18.6	2300.1 / 2300.2
**2**	H-TFFYGGSRGKRNNFK(Dau=Aoa)TEEY-OH	20.9	2882.3 / 2882.3
**3**	Dau=Aoa-TFFYGGSRGKRNNFKTEEY-OH	21.9	2882.3 / 2882.3
**4**	H-TFFYGGSRGK(Dau=Aoa)RNNFKTEEY-OH	21.2	2882.3 / 2882.3
**5**	Dau=Aoa-TFFYGGSRGKRNNFK(Dau=Aoa)TEEY-OH	21.8	3464.5 / 3464.5
**6**	H-TFFYGGSRGK(Dau=Aoa)RNNFK(Dau=Aoa)TEEY-OH	20.6	3464.5 / 3464.5
**7**	Dau=Aoa-TFFYGGSRGK(Dau=Aoa)RNNFKTEEY-OH	21.8	3464.5 / 3464.5
**8**	Dau=Aoa-TFFYGGSRGK(Dau=Aoa)RNNFK(Dau=Aoa)TEEY-OH	21.6	4046.7 / 4046.6

[a] Macherey-Nagel Nucleosil C18 column (5 µm, 100 Å, 250 × 4,6 mm); gradient: 0 min 2% B, 5 min 2% B, 30 min 90% B; eluent A: 0.1% TFA / distilled water, eluent B: 0.1% TFA / acetonitrile:distilled water (80:20, v/v); flow rate: 1 mL/min; detection: 214 nm.

[b] ESI-MS Bruker Daltonics Esquire 3000 Plus

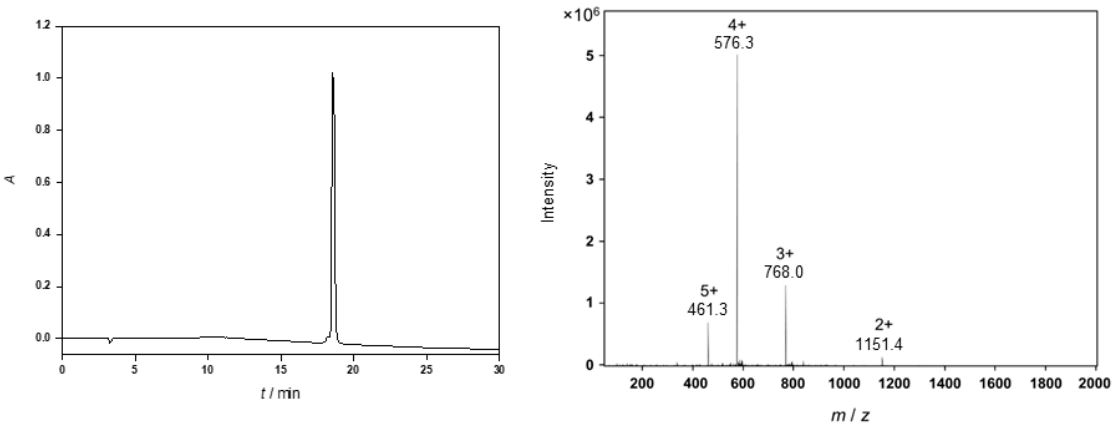

Figure S1. Analytical chromatogram and MS spectrum of Angiopep-2 (**1**)

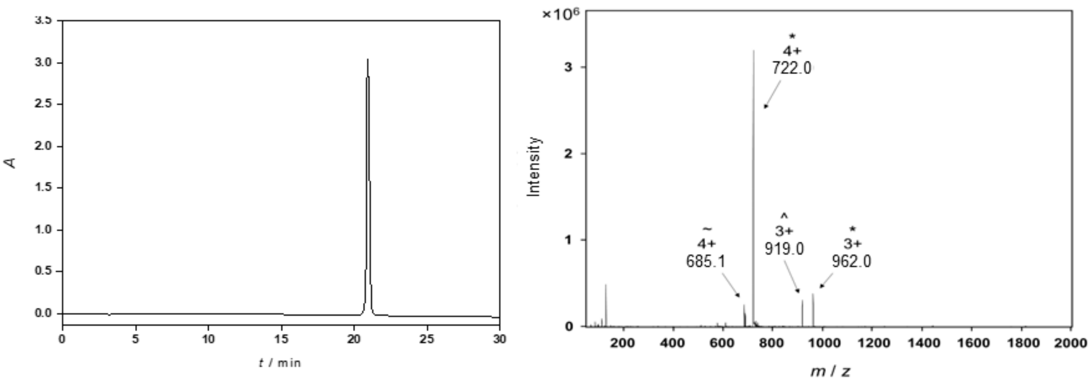

Figure S2. Analytical chromatogram and MS spectrum of H-TFFYGGSRGKRNNFK(Dau=Aoa)TEEY-OH (**2**). Peaks marked with ~ and ^ belong to the fragments resulting from the sugar loss of daunomycin occurring under general conditions of MS.

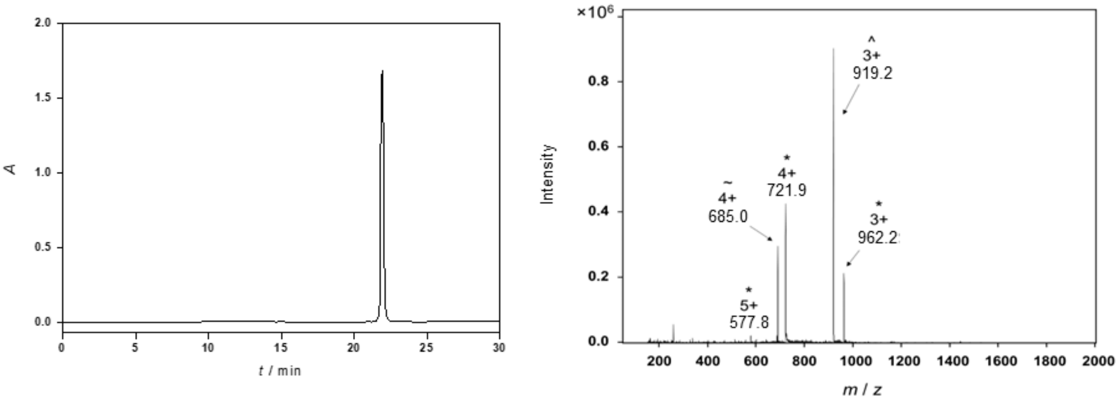

Figure S3. Analytical chromatogram and MS spectrum of Dau=Aoa-TFFYGGSRGKRNNFKTEEY-OH (**3**). Peaks marked with ~ and ^ belong to the fragments resulting from the sugar loss of daunomycin occurring under general conditions of MS.

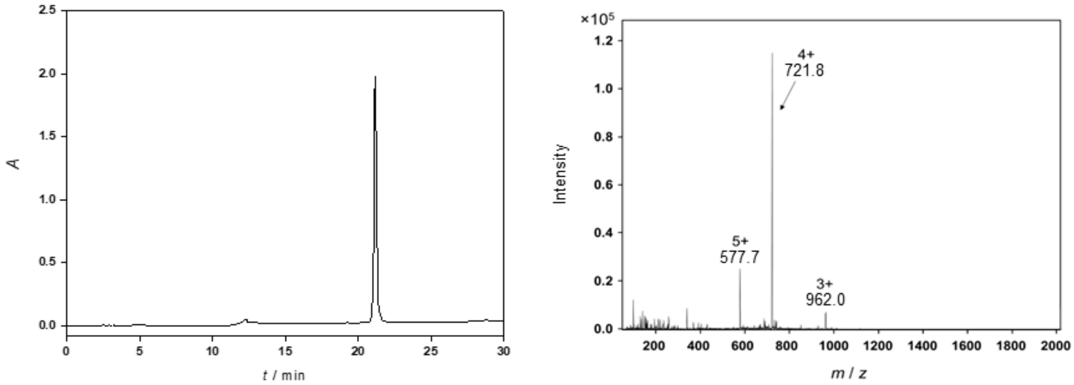

Figure S4. Analytical chromatogram and MS spectrum of H-TFFYGGSRGK(Dau=Aoa)RNNFKTEEY-OH (**4**)

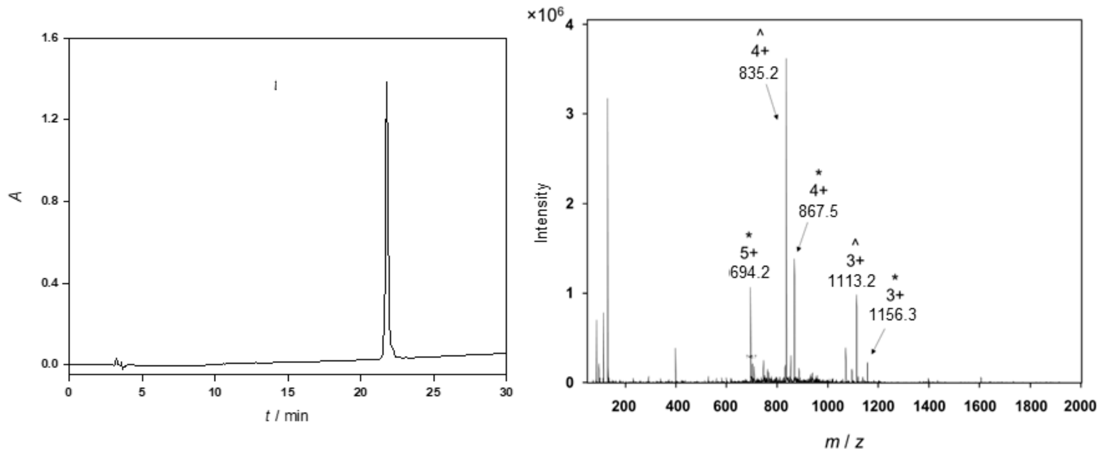

Figure S5. Analytical chromatogram and MS spectrum of Dau=Aoa-TFFYGGSRGKRNNFK(Dau=Aoa)TEEY-OH (**5**). Peaks marked with ^ belong to the fragments resulting from the sugar loss of daunomycin occurring under general conditions of MS.

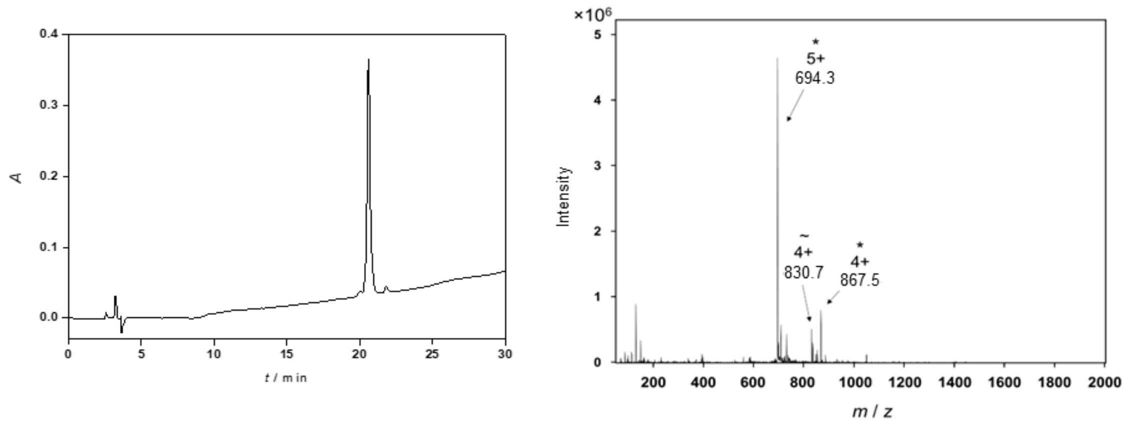

Figure S6. Analytical chromatogram and MS spectrum of H-TFFYGGSRG(Dau=Aoa)KRNNFK(Dau=Aoa)TEEY-OH (**6**). Peaks marked with ~ belong to the fragments resulting from the sugar loss of daunomycin occurring under general conditions of MS.

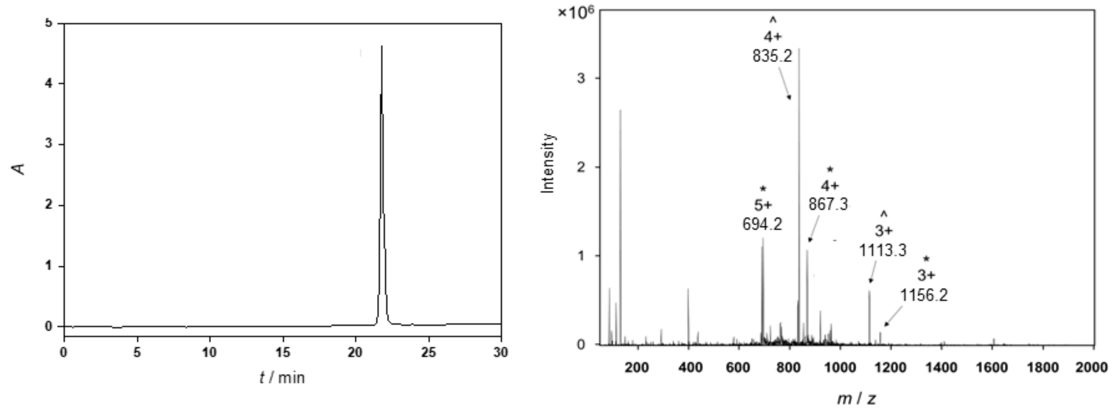

Figure S7. Analytical chromatogram and MS spectrum of Dau=Aoa-TFFYGGSRGK(Dau=Aoa)RNNFKTEEY-OH (**7**). Peaks marked with ^ belong to the fragments resulting from the sugar loss of daunomycin occurring under general conditions of MS.

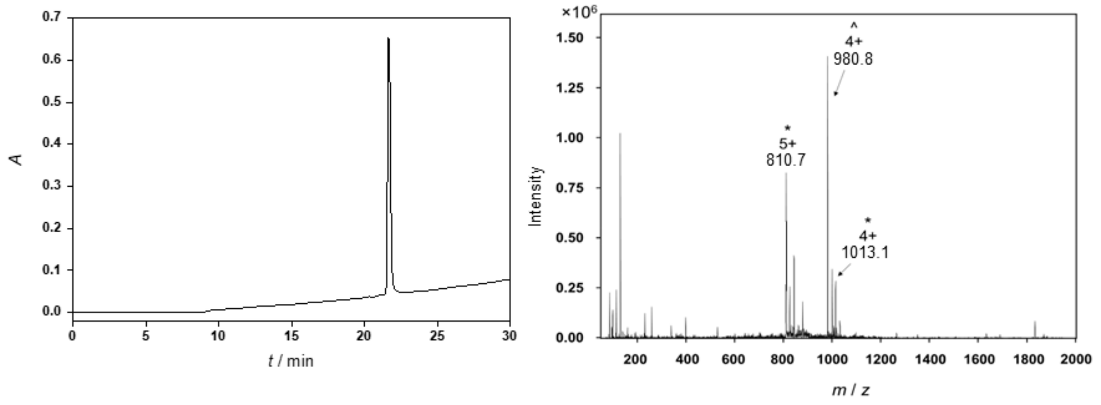

Figure S8. Analytical chromatogram and MS spectrum of Dau=Aoa-TFFYGGSRGK(Dau=Aoa)RNNFK(Dau=Aoa)TEEY-OH (**8**). Peaks marked with ^ belong to the fragments resulting from the sugar loss of daunomycin occurring under general conditions of MS.

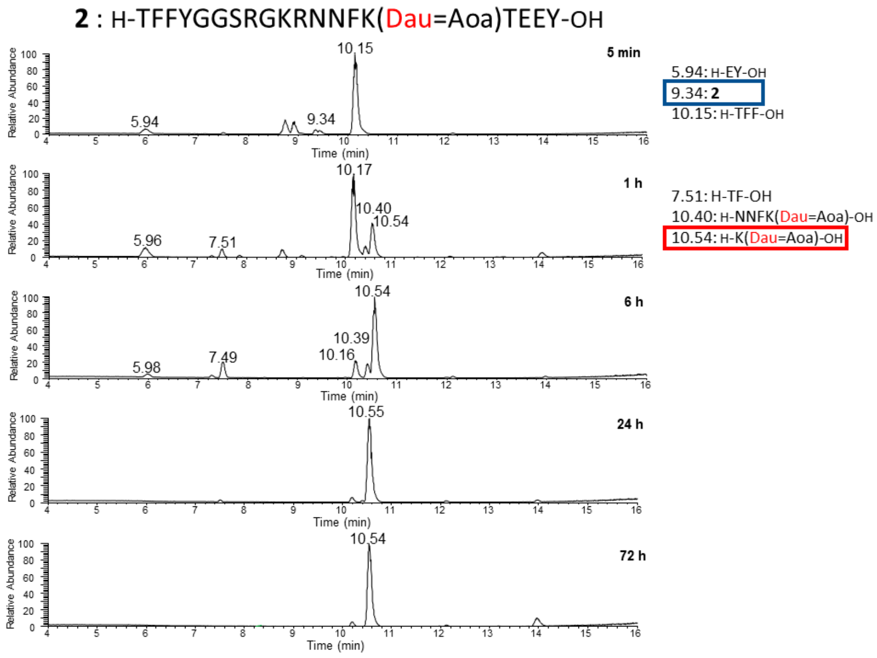

Figure S9. Detected total ion chromatograms showing the lysosomal degradation of conjugate **2**. (The blue box indicates the parent conjugate, while the red box marks the smallest drug containing metabolite.)

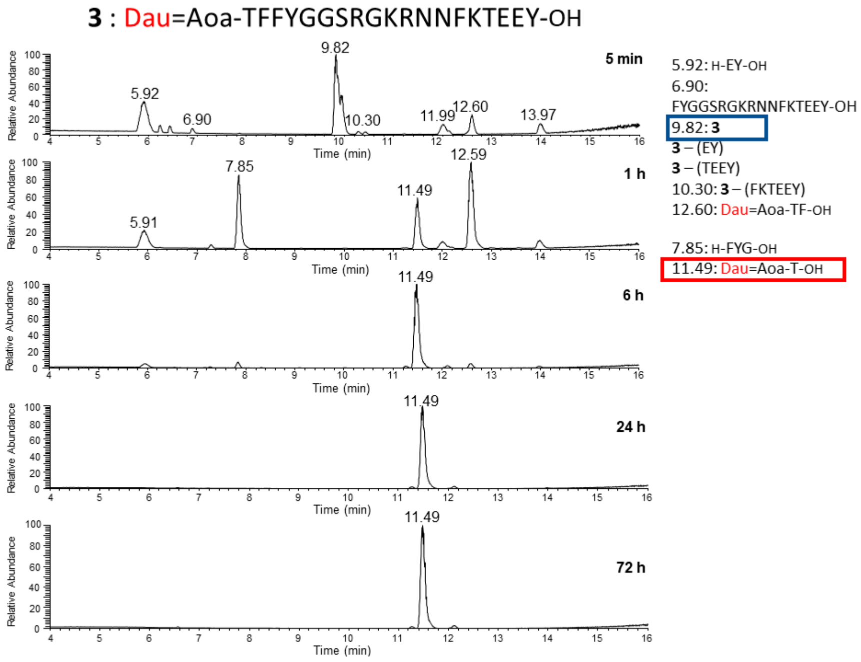

Figure S10. Detected total ion chromatograms showing the lysosomal degradation of conjugate **3**. (The blue box indicates the parent conjugate, while the red box marks the smallest drug containing metabolite.)

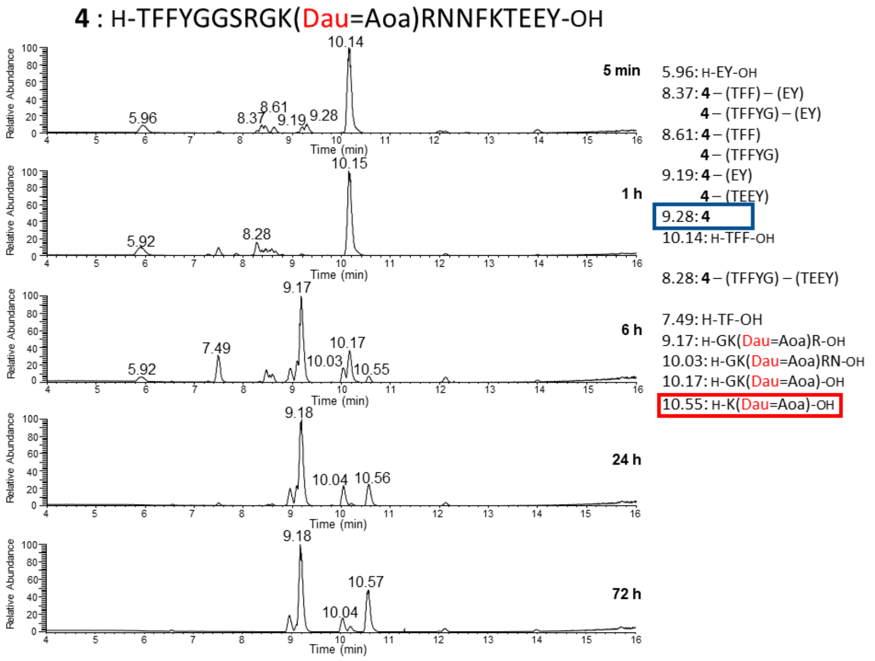

Figure S11. Detected total ion chromatograms showing the lysosomal degradation of conjugate **4**. (The blue box indicates the parent conjugate, while the red box marks the smallest drug containing metabolite.)

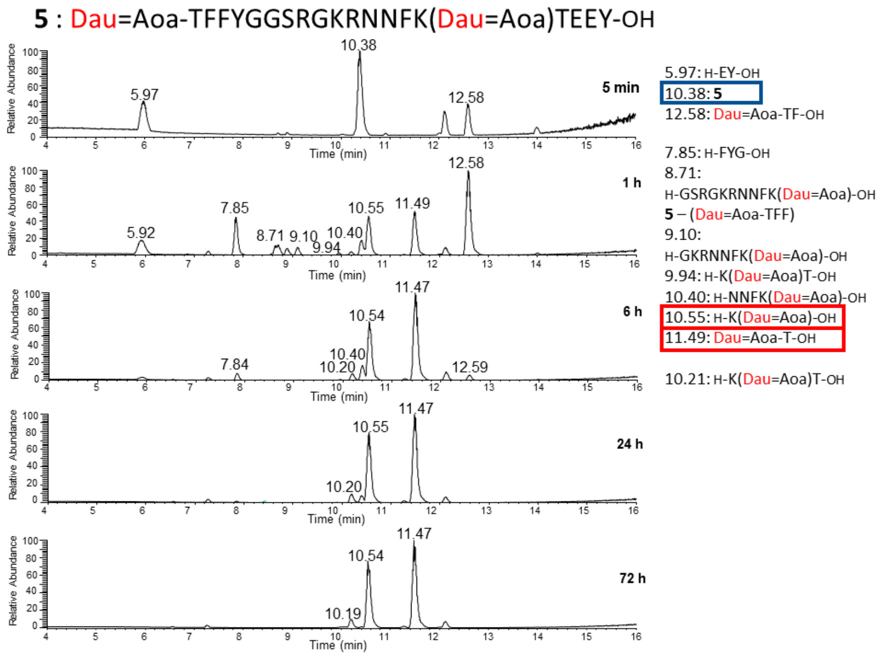

Figure S12. Detected total ion chromatograms showing the lysosomal degradation of conjugate **5**. (The blue box indicates the parent conjugate, while the red boxes mark the smallest drug containing metabolites.)

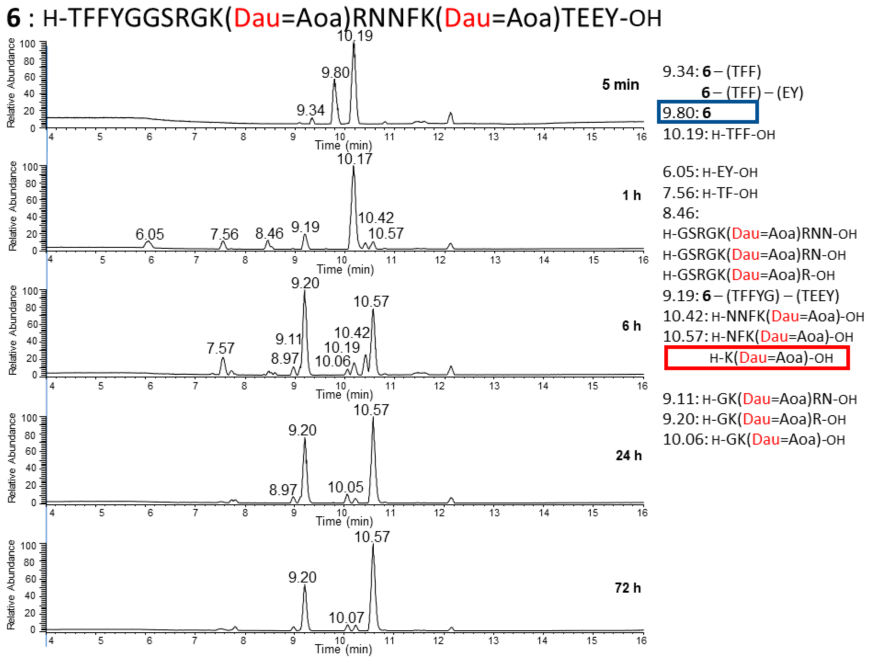

Figure S13. Detected total ion chromatograms showing the lysosomal degradation of conjugate **6**. (The blue box indicates the parent conjugate, while the red box marks the smallest drug containing metabolite.)

7 : Dau=Aoa-TFFYGGSRGK(Dau=Aoa)RNNFKTEEY-OH

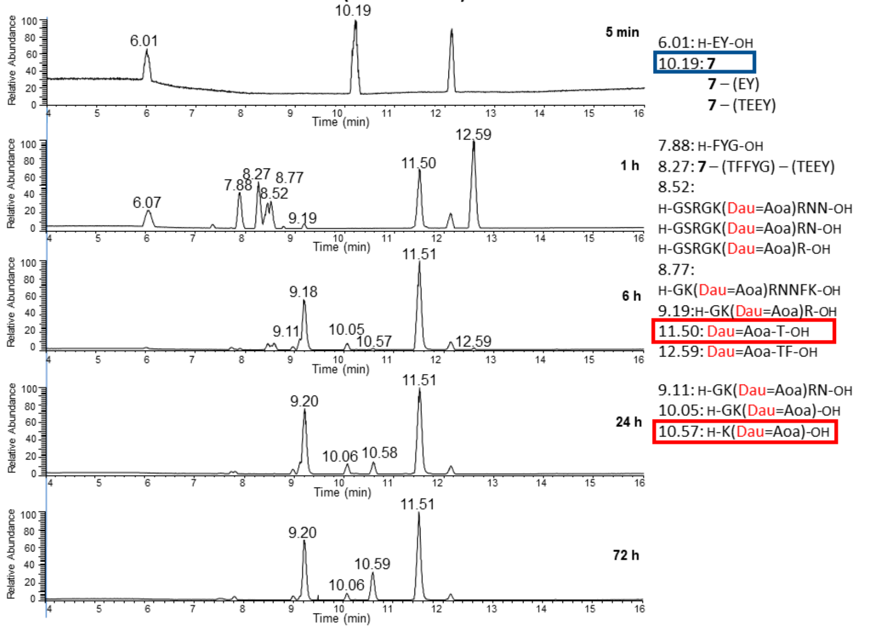

Figure S14. Detected total ion chromatograms showing the lysosomal degradation of conjugate **7**. (The blue box indicates the parent conjugate, while the red boxes mark the smallest drug containing metabolites.)

8 : Dau=Aoa-TFFYGGSRGK(Dau=Aoa)RNNFK(Dau=Aoa)TEEY-OH

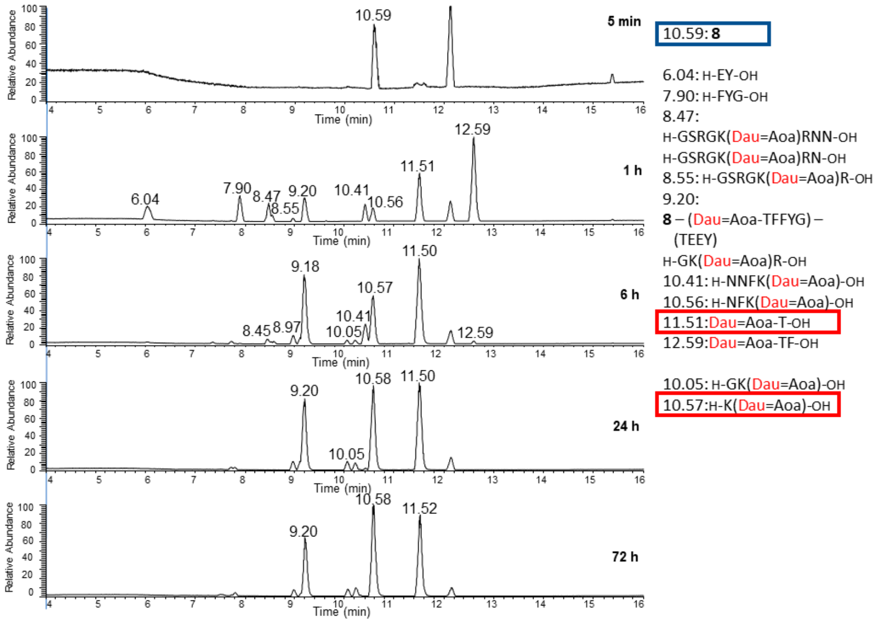

Figure S15. Detected total ion chromatograms showing the lysosomal degradation of conjugate **8**. (The blue box indicates the parent conjugate, while the red boxes mark the smallest drug containing metabolites.)

Table S2. Detected fragments from the lysosomal degradation of conjugate **2**.[a]

	Fragment	Chemical formula	MW_{calc}	MW_{meas}	Δ ppm
**1**	H-TFFYGGSRGKRNNFK(Dau=Aoa)TEEY-OH	$C_{133}H_{179}N_{31}O_{42}$	2882.2824	2882.2761	-2.19
**2**	H-TFFYGGSRGKRNNFK(Dau=Aoa)TE-OH	$C_{119}H_{163}N_{29}O_{37}$	2590.1765	2590.1682	-3.20
**3**	H-YGGSRGKRNNFK(Dau=Aoa)TEEY-OH	$C_{111}H_{154}N_{28}O_{38}$	2487.0979	2487.0928	-2.05
**4**	H-YGGSRGKRNNFK(Dau=Aoa)TE-OH	$C_{97}H_{138}N_{26}O_{33}$	2194.9920	2194.9866	-2.46
**5**	H-YGGSRGKRNNFK(Dau=Aoa)-OH	$C_{88}H_{124}N_{24}O_{28}$	1964.9017	1964.8953	-3.26
**6**	H-GKRNNFK(Dau=Aoa)-OH	$C_{66}H_{92}N_{16}O_{21}$	1444.6623	1444.6582	-2.84
**7**	H-NNFK(Dau=Aoa)-OH	$C_{52}H_{65}N_{9}O_{18}$	1103.4448	1103.4420	-2.54
**8**	H-K(Dau=Aoa)T-OH	$C_{39}H_{51}N_{5}O_{15}$	829.3382	829.3358	-2.89
**9**	H-K(Dau=Aoa)-OH	$C_{35}H_{44}N_{4}O_{13}$	728.2905	728.2878	-3.71
**10**	H-TFF-OH	$C_{22}H_{27}N_{3}O_{5}$	413.1951	413.1937	-3.39
**11**	H-EY-OH	$C_{14}H_{18}N_{2}O_{6}$	310.1165	310.1151	-4.51
**12**	H-TF-OH	$C_{13}H_{18}N_{2}O_{4}$	266.1267	266.1254	-4.88

[a] The grey row indicates the smallest drug containing metabolite.

Table S3. Detected fragments from the lysosomal degradation of conjugate **3**.[a]

	Fragment	Chemical formula	MW_{calc}	MW_{meas}	Δ ppm
**1**	Dau=Aoa-TFFYGGSRGKRNNFKTEEY-OH	$C_{133}H_{179}N_{31}O_{42}$	2882.2824	2882.2767	-1.98
**2**	Dau=Aoa-TFFYGGSRGKRNNFKTE-OH	$C_{119}H_{163}N_{29}O_{37}$	2590.1765	2590.1716	-1.89
**3**	Dau=Aoa-TFFYGGSRGKRNNFK-OH	$C_{110}H_{149}N_{27}O_{32}$	2360.0862	2360.0796	-2.80
**4**	Dau=Aoa-TFFYGGSRGKRNN-OH	$C_{95}H_{128}N_{24}O_{30}$	2084.9228	2084.9196	-1.53
**5**	H-FYGGSRGKRNNFKTEEY-OH	$C_{91}H_{133}N_{27}O_{28}$	2051.9813	2051.9760	-2.58
**6**	Dau=Aoa-TF-OH	$C_{42}H_{48}N_{4}O_{15}$	848.3116	848.3095	-2.48
**7**	Dau=Aoa-T-OH	$C_{33}H_{39}N_{3}O_{14}$	701.2432	701.2408	-3.42
**8**	H-FYG-OH	$C_{20}H_{23}N_{3}O_{5}$	385.1638	385.1624	-3.63
**9**	H-EY-OH	$C_{14}H_{18}N_{2}O_{6}$	310.1165	310.1152	-4.19

[a] The grey row indicates the smallest drug containing metabolite.

Table S4. Detected fragments from the lysosomal degradation of conjugate **4**.[a]

	Fragment	Chemical formula	MW$_{calc}$	MW$_{meas}$	Δ ppm
**1**	H-TFFYGGSRGK(Dau=Aoa)RNNFKTEEY-OH	$C_{133}H_{179}N_{31}O_{42}$	2882.2824	2882.2764	-2.08
**2**	H-TFFYGGSRGK(Dau=Aoa)RNNFKTE-OH	$C_{119}H_{163}N_{29}O_{37}$	2590.1765	2590.1694	-2.74
**3**	H-YGGSRGK(Dau=Aoa)RNNFKTEEY-OH	$C_{111}H_{154}N_{28}O_{38}$	2487.0979	2487.0903	-3.06
**4**	H-TFFYGGSRGK(Dau=Aoa)RNNFK-OH	$C_{110}H_{149}N_{27}O_{32}$	2360.0862	2360.0793	-2.92
**5**	H-GSRGK(Dau=Aoa)RNNFKTEEY-OH	$C_{100}H_{142}N_{26}O_{35}$	2267.0131	2267.0070	-2.69
**6**	H-YGGSRGK(Dau=Aoa)RNNFKTE-OH	$C_{97}H_{138}N_{26}O_{33}$	2194.9920	2194.9876	-2.00
**7**	H-YGGSRGK(Dau=Aoa)RNNFK-OH	$C_{88}H_{124}N_{24}O_{28}$	1964.9017	1964.8970	-2.39
**8**	H-GSRGK(Dau=Aoa)RNNFK-OH	$C_{77}H_{112}N_{22}O_{25}$	1744.8169	1744.8122	-2.69
**9**	H-GK(Dau=Aoa)RN-OH	$C_{47}H_{65}N_{11}O_{17}$	1055.4560	1055.4530	-2.84
**10**	H-GK(Dau=Aoa)R-OH	$C_{43}H_{59}N_{9}O_{15}$	941.4131	941.4102	-3.08
**11**	H-GK(Dau=Aoa)-OH	$C_{37}H_{47}N_{5}O_{14}$	785.3120	785.3096	-3.06
**12**	H-K(Dau=Aoa)-OH	$C_{35}H_{44}N_{4}O_{13}$	728.2905	728.2887	-2.47
**13**	H-TFF-OH	$C_{22}H_{27}N_{3}O_{5}$	413.1951	413.1937	-3.39
**14**	H-EY-OH	$C_{14}H_{18}N_{2}O_{6}$	310.1165	310.1152	-4.19
**15**	H-TF-OH	$C_{13}H_{18}N_{2}O_{4}$	266.1267	266.1255	-4.51

[a] The grey row indicates the smallest drug containing metabolite.

Table S5. Detected fragments from the lysosomal degradation of conjugate **5**.[a]

	Fragment	Chemical formula	MW_{calc}	MW_{meas}	Δ ppm
**1**	Dau=Aoa-TFFYGGSRGKRNNFK(Dau=Aoa)TEEY-OH	$C_{162}H_{209}N_{33}O_{53}$	3464.4673	3464.4582	-2.63
**2**	H-YGGSRGKRNNFK(Dau=Aoa)-OH	$C_{88}H_{124}N_{24}O_{28}$	1964.9017	1964.8962	-2.80
**3**	H-GSRGKRNNFK(Dau=Aoa)-OH	$C_{77}H_{112}N_{22}O_{25}$	1744.8169	1744.8106	-3.61
**4**	H-GKRNNFK(Dau=Aoa)-OH	$C_{66}H_{92}N_{16}O_{21}$	1444.6623	1444.6576	-3.25
**5**	H-NNFK(Dau=Aoa)-OH	$C_{52}H_{65}N_9O_{18}$	1103.4448	1103.4419	-2.63
**6**	Dau=Aoa-TF-OH	$C_{42}H_{48}N_4O_{15}$	848.3116	848.3092	-2.83
**7**	H-K(Dau=Aoa)T-OH	$C_{39}H_{51}N_5O_{15}$	829.3382	829.3361	-2.53
**8**	H-K(Dau=Aoa)-OH	$C_{35}H_{44}N_4O_{13}$	728.2905	728.2876	-3.98
**9**	Dau=Aoa-T-OH	$C_{33}H_{39}N_3O_{14}$	701.2432	701.2411	-2.99
**10**	H-FYG-OH	$C_{20}H_{23}N_3O_5$	385.1638	385.1623	-3.89
**11**	H-EY-OH	$C_{14}H_{18}N_2O_6$	310.1165	310.1151	-4.51

[a] The grey rows indicate the smallest drug containing metabolites.

Table S6. Detected fragments from the lysosomal degradation of conjugate **6**.[a]

	Fragment	Chemical formula	MW calc	MW meas	Δ ppm
**1**	H-TFFYGGSRGK(Dau=Aoa)RNNFK(Dau=Aoa)TEEY-OH	$C_{162}H_{209}N_{33}O_{53}$	3464.4673	3464.4564	-3.15
**2**	H-YGGSRGK(Dau=Aoa)RNNFK(Dau=Aoa)TEEY-OH	$C_{140}H_{184}N_{30}O_{49}$	3069.2828	3069.2739	-2.90
**3**	H-YGGSRGK(Dau=Aoa)RNNFK(Dau=Aoa)TE-OH	$C_{126}H_{168}N_{28}O_{44}$	2777.1769	2777.1666	-3.71
**4**	H-GSRGK(Dau=Aoa)RNNFK(Dau=Aoa)-OH	$C_{106}H_{142}N_{24}O_{36}$	2327.0019	2326.9952	-2.88
**5**	H-GSRGK(Dau=Aoa)RNN-OH	$C_{62}H_{91}N_{19}O_{23}$	1469.6535	1469.6480	-3.74
**6**	H-GSRGK(Dau=Aoa)RN-OH	$C_{58}H_{85}N_{17}O_{21}$	1355.6106	1355.6060	-3.39
**7**	H-GSRGK(Dau=Aoa)R-OH	$C_{54}H_{79}N_{15}O_{19}$	1241.5677	1241.5640	-2.98
**8**	H-NNFK(Dau=Aoa)-OH	$C_{52}H_{65}N_{9}O_{18}$	1103.4447	1103.4414	-2.99
**9**	H-GK(Dau=Aoa)RN-OH	$C_{47}H_{65}N_{11}O_{17}$	1055.4560	1055.4522	-3.60
**10**	H-NFK(Dau=Aoa)-OH	$C_{48}H_{59}N_{7}O_{16}$	989.4018	989.3988	-3.03
**11**	H-GK(Dau=Aoa)R-OH	$C_{43}H_{59}N_{9}O_{15}$	941.4131	941.4094	-3.93
**12**	H-GK(Dau=Aoa)-OH	$C_{37}H_{47}N_{5}O_{14}$	785.3120	785.3088	-4.07
**13**	H-K(Dau=Aoa)-OH	$C_{35}H_{44}N_{4}O_{13}$	728.2905	728.2880	-3.43
**14**	H-TFF-OH	$C_{22}H_{27}N_{3}O_{5}$	413.1951	413.1930	-5.08
**15**	H-EY-OH	$C_{14}H_{18}N_{2}O_{6}$	310.1165	310.1149	-5.16
**16**	H-TF-OH	$C_{13}H_{18}N_{2}O_{4}$	266.1267	266.1253	-5.26

[a] The grey row indicates the smallest drug containing metabolite.

Table S7. Detected fragments from the lysosomal degradation of conjugate **7**.[a]

	Fragment	Chemical formula	MW_{calc}	MW_{meas}	Δ ppm
**1**	Dau=Aoa-TFFYGGSRGK(Dau=Aoa)RNNFKTEEY-OH	$C_{162}H_{209}N_{33}O_{53}$	3464.4673	3464.4546	-3.67
**2**	Dau=Aoa-TFFYGGSRGK(Dau=Aoa)RNNFKTE-OH	$C_{148}H_{193}N_{31}O_{48}$	3172.3614	3172.3488	-3.97
**3**	Dau=Aoa-TFFYGGSRGK(Dau=Aoa)RNNFK-OH	$C_{139}H_{179}N_{29}O_{43}$	2942.2712	2942.2614	-3.33
**4**	H-GSRGK(Dau=Aoa)RNNFK-OH	$C_{77}H_{112}N_{22}O_{25}$	1744.8169	1744.8093	-4.36
**5**	H-GSRGK(Dau=Aoa)RNN-OH	$C_{62}H_{91}N_{19}O_{23}$	1469.6535	1469.6476	-4.01
**6**	H-GK(Dau=Aoa)RNNFK-OH	$C_{66}H_{92}N_{16}O_{21}$	1444.6623	1444.6576	-3.25
**7**	H-GSRGK(Dau=Aoa)RN-OH	$C_{58}H_{85}N_{17}O_{21}$	1355.6106	1355.6052	-3.98
**8**	H-GSRGK(Dau=Aoa)R-OH	$C_{54}H_{79}N_{15}O_{19}$	1241.5677	1241.5628	-3.95
**9**	H-GK(Dau=Aoa)RN-OH	$C_{47}H_{65}N_{11}O_{17}$	1055.4560	1055.4522	-3.60
**10**	H-GK(Dau=Aoa)R-OH	$C_{43}H_{59}N_{9}O_{15}$	941.4131	941.4101	-3.19
**11**	H-GK(Dau=Aoa)-OH	$C_{37}H_{47}N_{5}O_{14}$	785.3120	785.3090	-3.82
**12**	Dau=Aoa-TF-OH	$C_{42}H_{48}N_{4}O_{15}$	848.3116	848.3084	-3.77
**13**	H-K(Dau=Aoa)-OH	$C_{35}H_{44}N_{4}O_{13}$	728.2905	728.2880	-3.43
**14**	Dau=Aoa-T-OH	$C_{33}H_{39}N_{3}O_{14}$	701.2432	701.2402	-4.28
**15**	H-FYG-OH	$C_{20}H_{23}N_{3}O_{5}$	385.1638	385.1619	-4.93
**16**	H-EY-OH	$C_{14}H_{18}N_{2}O_{6}$	310.1165	310.1148	-5.48

[a] The grey rows indicate the smallest drug containing metabolites.

Table S8. Detected fragments from the lysosomal degradation of conjugate **8**.[a]

	Fragment	Chemical formula	MW_{calc}	MW_{meas}	Δ ppm
**1**	Dau=Aoa-TFFYGGSRGK(Dau=Aoa)RNNFK(Dau=Aoa)TEEY-OH	$C_{191}H_{239}N_{35}O_{64}$	4046.6523	4046.6460	-1.56
**2**	H-GSRGK(Dau=Aoa)RNNFK(Dau=Aoa)-OH	$C_{106}H_{142}N_{24}O_{36}$	2327.0019	2326.9950	-2.97
**3**	H-GSRGK(Dau=Aoa)RNN-OH	$C_{62}H_{91}N_{19}O_{23}$	1469.6535	1469.6478	-3.88
**4**	H-GSRGK(Dau=Aoa)RN-OH	$C_{58}H_{85}N_{17}O_{21}$	1355.6106	1355.6058	-3.54
**5**	H-GSRGK(Dau=Aoa)R-OH	$C_{54}H_{79}N_{15}O_{19}$	1241.5677	1241.5634	-3.46
**6**	H-NNFK(Dau=Aoa)-OH	$C_{52}H_{65}N_{9}O_{18}$	1103.4447	1103.4409	-3.44
**7**	H-NFK(Dau=Aoa)-OH	$C_{48}H_{59}N_{7}O_{16}$	989.4018	989.3984	-3.44
**8**	H-GK(Dau=Aoa)R-OH	$C_{43}H_{59}N_{9}O_{15}$	941.4131	941.4094	-3.93
**9**	Dau=Aoa-TF-OH	$C_{42}H_{48}N_{4}O_{15}$	848.3116	848.3082	-4.01
**10**	H-GK(Dau=Aoa)-OH	$C_{37}H_{47}N_{5}O_{14}$	785.3120	785.3090	-3.82
**11**	H-K(Dau=Aoa)-OH	$C_{35}H_{44}N_{4}O_{13}$	728.2905	728.2877	-3.84
**12**	Dau=Aoa-T-OH	$C_{33}H_{39}N_{3}O_{14}$	701.2432	701.2401	-4.42
**13**	H-FYG-OH	$C_{20}H_{23}N_{3}O_{5}$	385.1638	385.1619	-4.93
**14**	H-EY-OH	$C_{14}H_{18}N_{2}O_{6}$	310.1165	310.1149	-5.16

[a] The grey rows indicate the smallest drug containing metabolites.

Table S9. Analytical data of the enzyme labile spacer containing Angiopep-2 – daunomycin conjugates

Code	Conjugate	R_t / min[a]	MW_{calc} / MW_{meas}[b]
**9**	H-TFFYGGSRGK(Dau=Aoa-GFLG)RNNFKTEEY-OH	22.3	3256.5 / 3256.5
**10**	H-TFFYGGSRGK(Dau=Aoa-VA)RNNFKTEEY-OH	21.5	3052.4 / 3052.4
**11**	H-TFFYGGSRGK(Dau=Aoa-VAGG)RNNFKTEEY-OH	21.5	3166.4 / 3166.4

[a] Macherey-Nagel Nucleosil C18 column (5 µm, 100 Å, 250 × 4,6 mm); gradient: 0 min 2% B, 5 min 2% B, 30 min 90% B; eluent A: 0.1% TFA / distilled water, eluent B: 0.1% TFA / acetonitrile:distilled water (80:20, v/v); flow rate: 1 mL/min; detection: 214 nm.

[b] ESI-MS Bruker Daltonics Esquire 3000 Plus

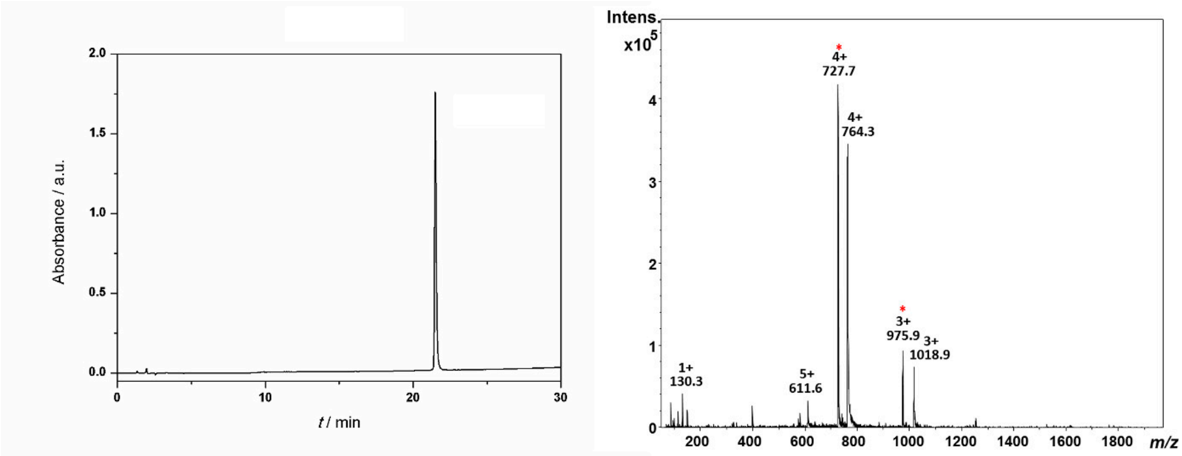

Figure S16. Analytical chromatogram and MS spectrum of H-TFFYGGSRGK(Dau=Aoa-GFLG)RNNFKTEEY-OH (**9**). Peaks marked with * belong to the fragments resulting from the sugar loss of daunomycin occurring under general conditions of MS.

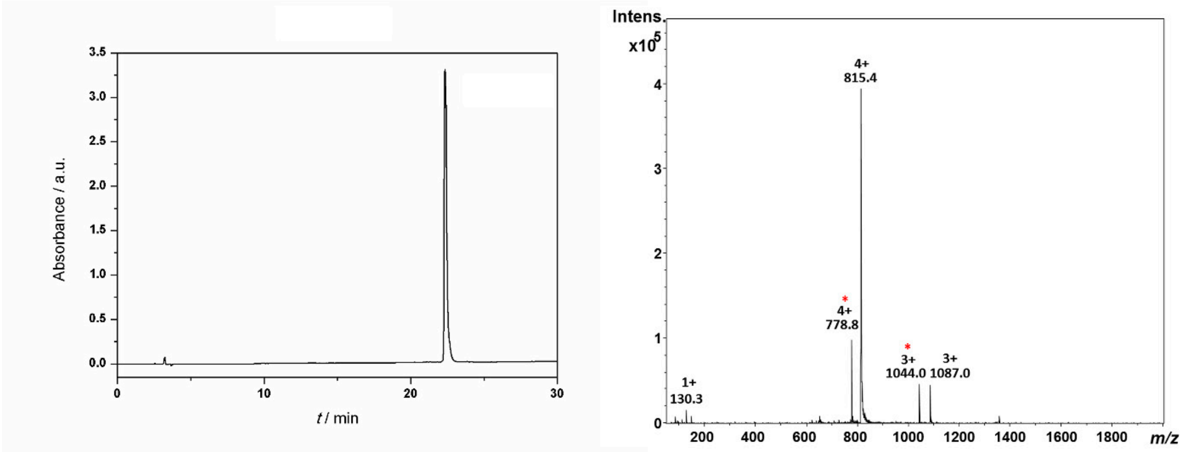

Figure S17. Analytical chromatogram and MS spectrum of H-TFFYGGSRGK(Dau=Aoa-VA)RNNFKTEEY-OH (**10**). Peaks marked with * belong to the fragments resulting from the sugar loss of daunomycin occurring under general conditions of MS.

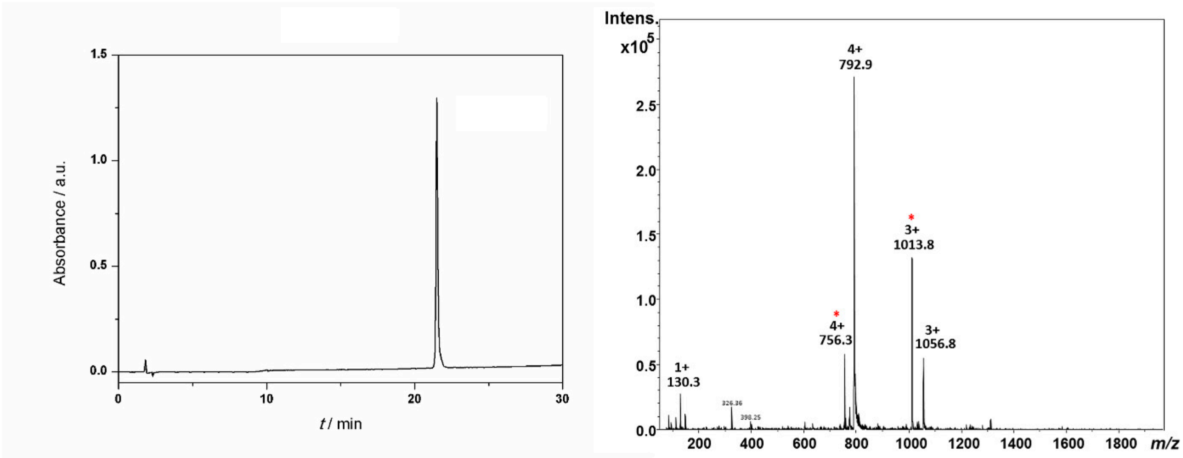

Figure S18. Analytical chromatogram and MS spectrum of H-TFFYGGSRGK(Dau=Aoa-VAGG)RNNFKTEEY-OH (**11**). Peaks marked with * belong to the fragments resulting from the sugar loss of daunomycin occurring under general conditions of MS.

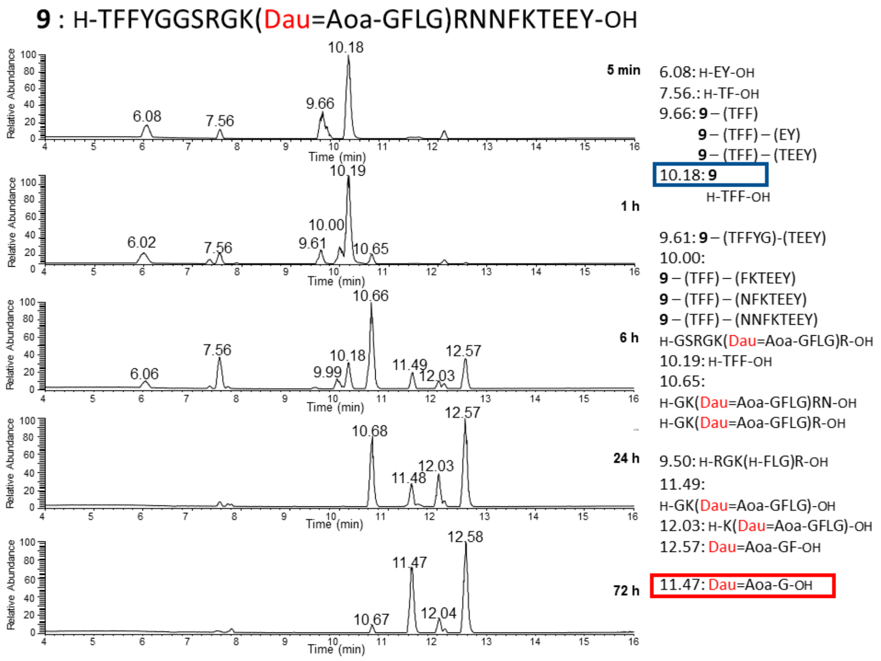

Figure S19. Detected total ion chromatograms showing the lysosomal degradation of conjugate **9**. (The blue box indicates the parent conjugate, while the red box marks the smallest drug containing metabolite.)

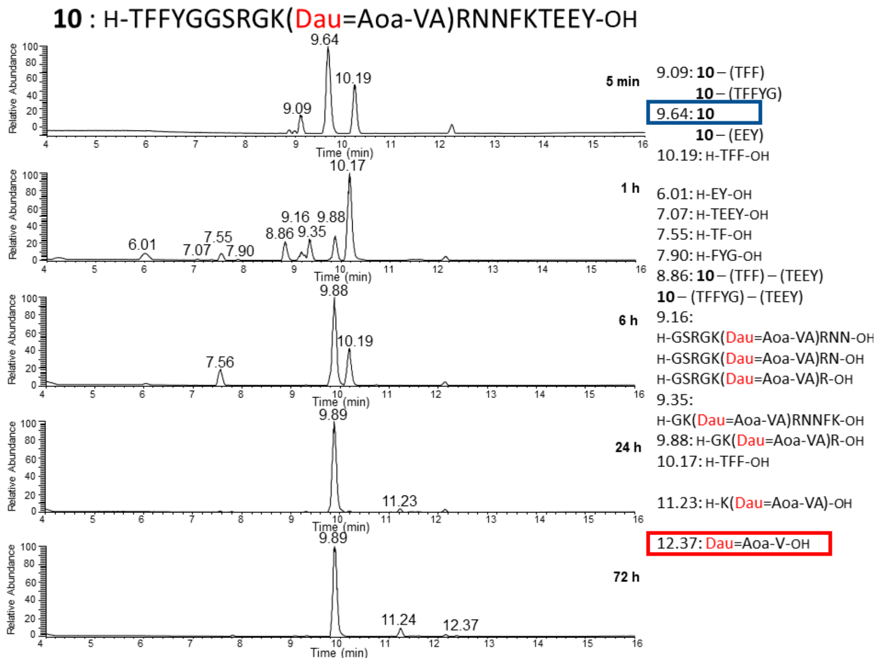

Figure S20. Detected total ion chromatograms showing the lysosomal degradation of conjugate **10**. (The blue box indicates the parent conjugate, while the red box marks the smallest drug containing metabolite.)

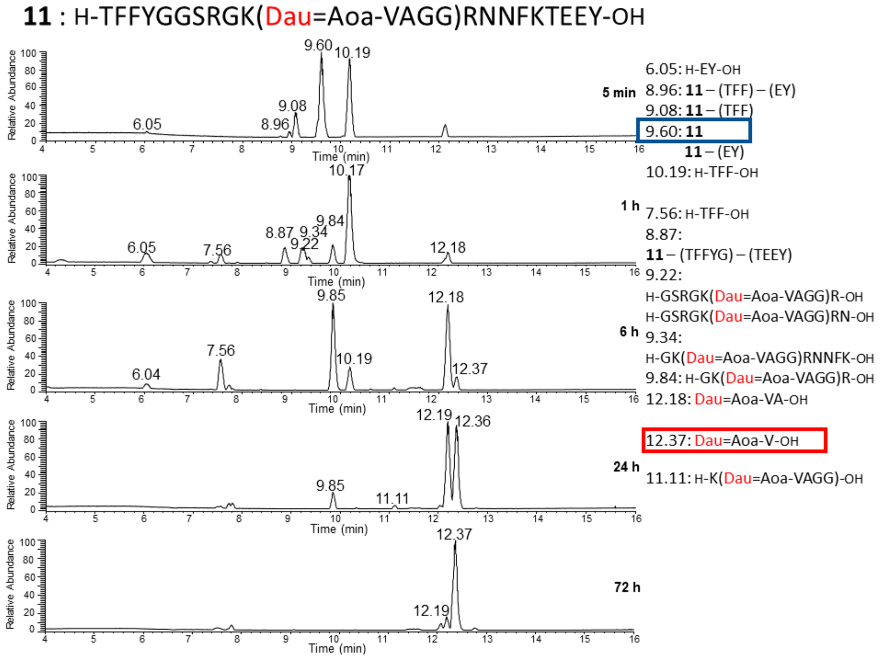

Figure S21. Detected total ion chromatograms showing the lysosomal degradation of conjugate **11**. (The blue box indicates the parent conjugate, while the red box marks the smallest drug containing metabolite.)

Table S10. Detected fragments from the lysosomal degradation of conjugate **9**.[a]

	Fragment	Chemical formula	MWcalc	MWmeas	Δ ppm
**1**	H-TFFYGGSRGK(Dau=Aoa-GFLG)RNNFKTEEY-OH	$C_{152}H_{205}N_{35}O_{46}$	3256.4778	3256.4668	-3.38
**2**	H-YGGSRGK(Dau=Aoa-GFLG)RNNFKTEEY-OH	$C_{130}H_{180}N_{32}O_{42}$	2861.2933	2861.2815	-4.12
**3**	H-YGGSRGK(Dau=Aoa-GFLG)RNNFKTE-OH	$C_{116}H_{164}N_{30}O_{37}$	2569.1874	2569.1775	-3.85
**4**	H-YGGSRGK(Dau=Aoa-GFLG)RNNFK-OH	$C_{107}H_{150}N_{28}O_{32}$	2339.0971	2339.0868	-4.40
**5**	H-GSRGK(Dau=Aoa-GFLG)RNNFK-OH	$C_{96}H_{138}N_{26}O_{29}$	2119.0123	2119.0026	-4.58
**6**	H-YGGSRGK(Dau=Aoa-GFLG)RNN-OH	$C_{92}H_{129}N_{25}O_{30}$	2063.9337	2063.9258	-3.83
**7**	H-YGGSRGK(Dau=Aoa-GFLG)RN-OH	$C_{88}H_{123}N_{23}O_{28}$	1949.8908	1949.8828	-4.10
**8**	H-YGGSRGK(Dau=Aoa-GFLG)R-OH	$C_{84}H_{117}N_{21}O_{26}$	1835.8479	1835.8404	-4.09
**9**	H-GGSRGK(Dau=Aoa-GFLG)RN-OH	$C_{79}H_{114}N_{22}O_{26}$	1786.8275	1786.8196	-4.42
**10**	H-GGSRGK(Dau=Aoa-GFLG)R-OH	$C_{75}H_{108}N_{20}O_{24}$	1672.7845	1672.7776	-4.12
**11**	H-GSRGK(Dau=Aoa-GFLG)R-OH	$C_{73}H_{105}N_{19}O_{23}$	1615.7631	1615.7556	-4.64
**12**	H-GK(Dau=Aoa-GFLG)RN-OH	$C_{66}H_{91}N_{15}O_{21}$	1429.6514	1429.6454	-4.20
**13**	H-GK(Dau=Aoa-GFLG)R-OH	$C_{62}H_{85}N_{13}O_{19}$	1315.6085	1315.6038	-3.57
**14**	H-GK(Dau=Aoa-GFLG)-OH	$C_{56}H_{73}N_{9}O_{18}$	1159.5074	1159.5041	-2.85
**15**	H-K(Dau=Aoa-GFLG)-OH	$C_{54}H_{70}N_{8}O_{17}$	1102.4859	1102.4827	-2.90
**16**	Dau=Aoa-GF-OH	$C_{40}H_{44}N_{4}O_{14}$	804.2854	804.2826	-3.48
**17**	Dau=Aoa-G-OH	$C_{31}H_{35}N_{3}O_{13}$	657.2170	657.2145	-3.80
**18**	H-TFF-OH	$C_{22}H_{27}N_{3}O_{5}$	413.1951	413.1931	-4.84
**19**	H-EY-OH	$C_{14}H_{18}N_{2}O_{6}$	310.1165	310.1148	-5.48
**20**	H-TF-OH	$C_{13}H_{18}N_{2}O_{4}$	266.1267	266.1253	-5.26

[a] The grey row indicates the smallest drug containing metabolite.

Table S11. Detected fragments from the lysosomal degradation of conjugate **10**.[a]

	Fragment	Chemical formula	MW_{calc}	MW_{meas}	Δ ppm
**1**	H-TFFYGGSRGK(Dau=Aoa-VA)RNNFKTEEY-OH	$C_{141}H_{193}N_{33}O_{44}$	3052.3879	3052.3761	-3.87
**2**	H-TFFYGGSRGK(Dau=Aoa-VA)RNNFKTE-OH	$C_{127}H_{177}N_{31}O_{39}$	2760.2820	2760.2706	-4.13
**3**	H-YGGSRGK(Dau=Aoa-VA)RNNFKTEEY-OH	$C_{119}H_{168}N_{30}O_{40}$	2657.2034	2657.1939	-3.58
**4**	H-GSRGK(Dau=Aoa-VA)RNNFKTEEY-OH	$C_{108}H_{156}N_{28}O_{37}$	2437.1186	2437.1088	-4.02
**5**	H-YGGSRGK(Dau=Aoa-VA)RNNFK-OH	$C_{96}H_{138}N_{26}O_{30}$	2135.0072	2134.9980	-4.31
**6**	H-GSRGK(Dau=Aoa-VA)RNNFK-OH	$C_{85}H_{126}N_{24}O_{27}$	1914.9224	1914.9144	-4.18
**7**	H-GSRGK(Dau=Aoa-VA)RNN-OH	$C_{70}H_{105}N_{21}O_{25}$	1639.7590	1639.7522	-4.15
**8**	H-GK(Dau=Aoa-VA)RNNFK-OH	$C_{74}H_{106}N_{18}O_{23}$	1614.7678	1614.7622	-3.47
**9**	H-GSRGK(Dau=Aoa-VA)RN-OH	$C_{66}H_{99}N_{19}O_{23}$	1525.7161	1525.7100	-4.00
**10**	H-GSRGK(Dau=Aoa-VA)R-OH	$C_{62}H_{93}N_{17}O_{21}$	1411.6732	1411.6678	-3.83
**11**	H-GK(Dau=Aoa-VA)R-OH	$C_{51}H_{73}N_{11}O_{17}$	1111.5186	1111.5147	-3.51
**12**	H-K(Dau=Aoa-VA)-OH	$C_{43}H_{58}N_{6}O_{15}$	898.3960	898.3929	-3.45
**13**	Dau=Aoa-V-OH	$C_{34}H_{41}N_{3}O_{13}$	699.2639	699.2615	-3.43
**14**	H-TEEY-OH	$C_{23}H_{32}N_{4}O_{11}$	540.2068	540.2051	-3.15
**15**	H-TFF-OH	$C_{22}H_{27}N_{3}O_{5}$	413.1951	413.1931	-4.84
**16**	H-FYG-OH	$C_{20}H_{23}N_{3}O_{5}$	385.1638	385.1624	-3.63
**17**	H-EY-OH	$C_{14}H_{18}N_{2}O_{6}$	310.1165	310.1149	-5.16
**18**	H-TF-OH	$C_{13}H_{18}N_{2}O_{4}$	266.1267	266.1253	-5.26

[a] The grey row indicates the smallest drug containing metabolite.

Table S12. Detected fragments from the lysosomal degradation of conjugate **11**.[a]

	Fragment	Chemical formula	MW_{calc}	MW_{meas}	Δ ppm
**1**	H-TFFYGGSRGK(Dau=Aoa-VAGG)RNNFKTEEY-OH	$C_{145}H_{199}N_{35}O_{46}$	3166.4308	3166.4211	-3.06
**2**	H-TFFYGGSRGK(Dau=Aoa-VAGG)RNNFKTE-OH	$C_{131}H_{183}N_{33}O_{41}$	2874.3249	2874.3138	-3.86
**3**	H-YGGSRGK(Dau=Aoa-VAGG)RNNFKTEEY-OH	$C_{123}H_{174}N_{32}O_{42}$	2771.2463	2771.2365	-3.54
**4**	H-YGGSRGK(Dau=Aoa-VAGG)RNNFKTE-OH	$C_{109}H_{158}N_{30}O_{37}$	2479.1404	2479.1313	-3.67
**5**	H-GSRGK(Dau=Aoa-VAGG)RNNFK-OH	$C_{89}H_{132}N_{26}O_{29}$	2028.9654	2028.9580	-3.65
**6**	H-GK(Dau=Aoa-VAGG)RNNFK-OH	$C_{78}H_{112}N_{20}O_{25}$	1728.8107	1728.8036	-4.11
**7**	H-GSRGK(Dau=Aoa-VAGG)RN-OH	$C_{70}H_{105}N_{21}O_{25}$	1639.7590	1639.7516	-4.51
**8**	H-GSRGK(Dau=Aoa-VAGG)R-OH	$C_{66}H_{99}N_{19}O_{23}$	1525.7161	1525.7096	-4.26
**9**	H-GK(Dau=Aoa-VAGG)R-OH	$C_{55}H_{79}N_{13}O_{19}$	1225.5615	1225.5562	-4.32
**10**	H-GK(Dau=Aoa-VAGG)-OH	$C_{49}H_{67}N_{9}O_{18}$	1069.4604	1069.4572	-2.99
**11**	H-K(Dau=Aoa-VAGG)-OH	$C_{47}H_{64}N_{8}O_{17}$	1012.4389	1012.4358	-3.06
**12**	Dau=Aoa-VA-OH	$C_{37}H_{46}N_{4}O_{14}$	770.3011	770.2986	-3.25
**13**	Dau=Aoa-V-OH	$C_{34}H_{41}N_{3}O_{13}$	699.2639	699.2613	-3.72
**14**	H-TFF-OH	$C_{22}H_{27}N_{3}O_{5}$	413.1951	413.1931	-4.84
**15**	H-EY-OH	$C_{14}H_{18}N_{2}O_{6}$	310.1165	310.1150	-4.84
**16**	H-TF-OH	$C_{13}H_{18}N_{2}O_{4}$	266.1267	266.1252	-5.64

[a] The grey row indicates the smallest drug containing metabolite.
